# A tuber mustard AP2/ERF transcription factor gene, *BjABR1*, functioning in abscisic acid and abiotic stress responses, and evolutionary trajectory of the *ABR1* homologous genes in *Brassica* species

**DOI:** 10.7717/peerj.6071

**Published:** 2018-12-11

**Authors:** Liuxin Xiang, Chao Liu, Jingzhi Luo, Lin He, Yushan Deng, Jie Yuan, Chaofeng Wu, Yingfan Cai

**Affiliations:** 1 Chongqing University of Posts and Telecommunications, Chongqing Key Laboratory on Big Data for Bio Intelligence, School of Bioinformatics, School of Software Engineering, Chongqing, China; 2 Henan University, State Key Laboratory of Cotton Biology, Henan Key Laboratory of Plant Stress Biology, School of Life Sciences, Kaifeng, Henan, China

**Keywords:** Abiotic stress and abscisic acid responses, *Brassica* species, ABR1 homologous genes, Tuber mustard, *BjABR1*, Evolutionary trajectory

## Abstract

The AP2/ERF superfamily of transcription factors is one of the largest transcription factor families in plants and plays an important role in plant development processes and stress responses. In this study, *BjABR1*, an AP2/ERF superfamily gene, from tuber mustard (*Brassica juncea* var. *tumida* Tsen et Lee), sharing high amino acid sequence similarity with the *AtABR1* (*Arabidopsis thaliana* AP2-like abscisic acid repressor 1) gene, were performed functional research, and the *ABR1* homologous genes in *Brassica* species were identified and performed phylogenetic analysis. The promoter sequence of *BjABR1* contained many phytohormone- and stress-related cis-elements; ABA (abscisic acid) and abiotic stresses can induce *BjABR1* expression in tuber mustard; overexpression of *BjABR1* in *Arabidopsis* can alleviate plant sensitivity to ABA and salt and osmotic stresses, and the alleviation may be due to changes in stress/ABA-induced gene expression. These results indicated that *BjABR1* functions in ABA and abiotic stress responses. By BLAST searches against the genome database of five *Brassica* species (three diploids, *B. rapa*, *B. nigra*, and *B. oleracea*, and two allotetraploid, *B. juncea* and *B. napus*) using the protein sequence of *AtABR1*, 3, 3, 3, 6, and 5 *ABR1* homologous genes in *B. nigra*, *B. rapa*, *B. oleracea*, *B. juncea*, and *B. napus* were identified, respectively, and they shared high sequence similarity. By sequence analysis, annotation mistakes of the protein-coding regions of two *ABR1* homologous genes, *GSBRNA2T00134741001* and *BjuB007684*, were found and corrected. Then, the evolution analysis of these *ABR1* homologous genes showed that the ancestor of the three diploid species** had three *ABR1* homologous genes and each diploid** inherited all the three genes from their ancestor; then, allotetraploid *B. juncea* inherited all the six genes from *B. rapa* and *B. nigra* with no gene lost, while allotetraploid *B. napus* inherited all the three genes from *B. oleracea* and two genes from *B. rapa* with one gene lost, indicating that *ABR1* homologous genes possessed greater hereditary conservation in *Brassica* species. The *ABR1* homologous genes between *B. rapa* and *B. oleracea* shared much higher sequence similarity compared to that of *B. nigra* in diploid** species, indicating that *ABR1* homologous genes in *B. nigra* had experienced more rapid evolution, and *B. rapa* and *B. oleracea* may share closer relationship compared to *B. nigra*. Moreover, the spatial and temporal expression analysis of six *ABR1* homologous genes of tuber mustard showed that they possessed different expression models. These results imply that *ABR1* homologous genes are important to *Brassica* plants, and they may possess similar function in ABA and abiotic stress responses but play a role in different tissues and growing stages of plant. This study will provide the foundation to the functional research of *ABR1* homologous genes in the *Brassica* species and help to reveal and understand the evolution mechanisms of *Brassica* species.

## Introduction

The AP2/ERF (APETELLA2/Ethylene Responsive Element Binding Factor) superfamily of transcription factors is one of the largest transcription factor families in plants. This gene family was believed to be part of the plant kingdom only for a long time, but recent reports showed that the AP2/ERF superfamily members were present in various protists and ciliates (Licausi, Ohme-Takagi & Perata, 2013). The AP2/ERF superfamily contains at least one AP2/ERF domain consisting of about 60–70 highly conserved amino acids and involving in DNA binding (Ohmetakagi & Shinshi, 1995).

Based on sequence similarities and the number of AP2/ERF domains, the superfamily was further classified into four families: ERF, AP2, RAV (Related to ABI3/VP) and Soloist (Nakano et al., 2006; Liu et al., 2013; Lata et al., 2014; Matías-Hernández et al., 2014). Most of the genes containing a single AP2/ERF domain are classified into ERF family. Genes containing multiple AP2/ERF domains are classified into AP2 family. Members containing AP2 domain and B3-like DNA-binding domain are classified into RAV family (Matías-Hernández et al., 2014). Besides, there is a small group of genes with a highly diverged AP2 domain and gene structure from the ERF and RAV families classified into the Soloists (Sakuma et al., 2002).

According to the amino acid sequence of the DNA binding domain, the ERF family was separated into two subfamilies, the DREB/CBF (dehydration responsive element binding/C-repeat CRT binding transcription factors) subfamily and ERF subfamily (Sakuma et al., 2002). The DREB/CBF hereafter referred as DREB is a major member of AP2/ERF superfamily, which binds to DRE (A/GCCGAC) or CRT (TGGCCGAC) core cis-acting sequences in promoters (Yamaguchi-Shinozaki & Shinozaki, 1994; Agarwal et al., 2006), while the ERF binds to GCC (AGCCGCC) core cis-acting sequences (Ohmetakagi & Shinshi, 1995).

With the availability of more genomic data, genome wide identification of AP2/ERF superfamily members has been conducted in various plants, such as *Arabidopsis* (Nakano et al., 2006), rice (Nakano et al., 2006), maize (Liu et al., 2013), foxtail millet (Lata et al., 2014), Barley (Guo et al., 2016), cauliflower (Li et al., 2017), pepper (Jin et al., 2018). Moreover, the functions of AP2/ERF genes have been widely studied in the model plant *Arabidopsis* and in other plants. The AP2/ERF gene members play an important role in the regulation of different development processes as well as stress responses in plants. The AP2 family genes function mainly in plant development, such as in flower (Licausi, Ohme-Takagi & Perata, 2013; Aukerman & Sakai, 2003; Zhang et al., 2018), seed (Wang et al., 2016; Ohto et al., 2009), leaf (Moose & Sisco, 1996), root (Kitomi et al., 2011). The RAV family genes are considered to regulate plant development and participate in stress responses (Matías-Hernández et al., 2014; Fu et al., 2014). The DREB subfamily genes function mainly in abiotic stress responses, such as drought (Liu et al., 2013; Liao et al., 2016), heat (Qin et al., 2007; Hong et al., 2009), low-temperature (Sakuma et al., 2002), osmotic (Wang et al., 2011a), and high-salt stress (Schmidt et al., 2013). The ERF subfamily genes are characterized to play a role in stress responses (Müller & Munné-Bosch, 2015), plant hormone responses (Müller & Munné-Bosch, 2015; Pandey et al., 2005), plant metabolism, growth, and development (Go et al., 2014; Chandler et al., 2007; Nie et al., 2018), and so on. The Soloist genes are considered to play a role in hormone, biotic and abiotic responses (Giri et al., 2014).

The *Brassica* is an important genus of Brassicaceae and have rich diversity with respect to both speciation and the abundant morphotypes in each *Brassica* species. *Brassica* species are also important crops grown worldwide for human nutrition, containing a diverse range of oilseed, condiment, and vegetable crops (Warwick, Francis & Al-Shehbaz, 2006). The “triangle of U” model (Nagaharu, 1935) has been applied to describe the evolutionary relationships among the six widely cultivated *Brassica* species, the diploids *Brassica rapa* (AA), *B. nigra* (BB), and *B. oleracea* (CC) and the allotetraploids *B. juncea* (AABB), *B. napus* (AACC), and *B. carinata* (BBCC). *B. juncea*, *B. napus*, and *B. carinata* were formed by hybridization between *B. rapa* and *B. nigra*, between *B. rapa* and *B. oleracea*, and between *B. nigra* and *B. oleracea*, respectively, followed by spontaneous chromosome doubling. To this day, the genomes of the five important *Brassica* species, *B. rapa*, *B. oleracea*, *B. nigra*, *B. napus*, and *B. juncea* have been published (Wang et al., 2011b; Liu et al., 2014; Parkin et al., 2014; Chalhoub et al., 2014; Yang et al., 2016). The genome-wide analysis of the AP2_ERF superfamily genes has been conducted in *B. rapa*, *B. oleracea*, and *B. napus* (Song, Li & Hou, 2013; Li et al., 2017; Thamilarasan et al., 2014; Song et al., 2016).

Tuber mustard, *B. juncea* var. *tumida* Tsen et Lee, is a very popular vegetable used as raw material for Fuling mustard. In previous study, we have analyzed the transcriptome of stem development in tuber mustard by RNA sequencing (Sun et al., 2012), and then, we cloned one of differentially expressed genes, named *BjABR1* (GenBank accession No. JQ713825.1) (Xiang et al., 2014), which shared high amino acid sequence similarity with the *AtABR1* (*Arabidopsis thaliana* AP2-like abscisic acid repressor 1) gene (*AT5g64750*) (Pandey et al., 2005). *BjABR1* protein contained a conserved AP2/ERF domain and was located in nucleus by onion epidermal subcellular localization analysis, indicating that *BjABR1* was a potential AP2/ERF transcription factor gene (Xiang et al., 2014). The *AtABR1* gene of *A. thaliana* responded to ABA and stress conditions including cold, high salt, and drought, and functioned as a repressor of ABA response in *Arabidopsis* (Pandey et al., 2005). The expression of *BjABR1* in tuber mustard was inducible under salt, osmotic and cold stresses (Xiang et al., 2014). Here, we show data on *BjABR1* which functions in ABA and abiotic stress responses. Moreover, due to the availability of the genome data of the five *Brassica* species, *B. rapa*, *B. oleracea*, *B. nigra*, *B. napus*, and *B. juncea*, it is possible to study systematically a class of homologous genes in the *Brassica* species. Here, we present the evolutionary trajectory of *ABR1* homologous genes in *Brassica* species and the expression patterns of *ABR1* homologous genes in tuber mustard. This study will provide the foundation to the functional research of *ABR1* homologous genes in the *Brassica* species and help to reveal and understand the evolution mechanisms of *Brassica* species.

## Materials and Methods

### Plant growth and stress treatments to tuber mustard

Seeds of tuber mustard cultivar *Yong’an* were provided by Chongqing Fuling Institute of Agricultural Sciences, Chongqing, China. The seeds were sown in October and plants grew in their natural environments in Chongqing, China. The roots, stems, and leaf of 14-week-old (before the start of stem swelling) and 16-week-old (stem welling stage) tuber mustard were respectively harvested and frozen immediately in liquid nitrogen for total RNA preparation and semi-quantitative analysis. For ABA treatment, abscisic acid (ABA, 10 µM) were sprayed onto 1-month-old tuber mustard seedlings to ensure total coverage of the foliage area. The seedlings were collected respectively at 0, 2, 4, 6, and 12 h after ABA treatments with three biological replicates, and frozen immediately in liquid nitrogen for total RNA preparation and qRT-PCR analysis.

### Cloning of *BjABR1* promoter and sequence analysis

The tuber mustard genomic DNA were used for genome walking according to the described method previously (Li et al., 2002). The TAIL-PCR method and the Genome Walking kit (TaKaRa, Dalian, China) were used to perform genome walking to isolate the promoter region of *BjABR1* gene following the manufacturer’s protocol. The random primers were improved following a previous study (Liu & Chen, 2007), and the three specific primers, SP1 (5′-AAGGAGGCGTGAGGGAGTT-3′), SP2 (5′-TCACGGTGGAGGTAGTCATC-3′), and SP3 (5′-TTCCTGATTTGCCACTTTT-3′), were designed as reverse primers according to the genomic DNA sequence of *BjABR1*. The PLANTCARE database (http://bioinformatics.psb.ugent.be/webtools/plantcare/html/) was used to identify potential cis-regulatory elements within the promoter.

### Construction of the plant expression vector and generation of transgenic plants

To generate the plant transformation vector pCAMBIA1302::*BjABR1*, the cDNA fragment with the complete ORF of *BjABR1* was synthesized from the total RNA using a pair of primers (sense primer: 5′- AGATCT (*Bal*II) TGCGTGCCTTAAAAGTG -3′, reverse primer: 5′-GGTTACC (*Bst E*II) TTAAGAGGATGGGCTAT -3′). After validating its sequence, the PCR fragments were digested by *Bal*II and *Bst E*II and ligated into the plasmid pCAMBIA1302 expression vectors which were also digested by *Bal*II and *Bst E*II but removed the GFP gene. The pCAMBIA1302::*BjABR1* constructs were then transferred into *Agrobacterium tumefaciens* GV3101 cells.

*Arabidopsis thaliana* (ecotype Col-0) seeds were sown on Murashige and Skoog (MS) medium containing 1% sucrose and 1% agar in a growth incubator. The growth conditions were as below: 23 °C, 75% relative humidity, and the photoperiod of 16 h light and 8 h darkness. The pCAMBIA1302::*BjABR1* constructs were transferred into *Arabidopsis* plants using the floral dip method to obtain ﬁrst-generation (T0) seeds. To obtain the homozygous, T0 seeds were planted on MS medium with 25 mg L^−1^ hygromycin (Dingguo, Beijing, China) to obtain second-generation (T1) seeds. The T1 seeds were then planted on MS medium with 25 mg L^−1^ hygromycin, and the transgenic lines with a segregation ratio of 3:1 (resistant:sensitive) were selected to be third-generation (T2) seeds. The T2 seeds were planted on MS medium with 25 mg L^−1^ hygromycin. When the T3 lines displayed 100% hygromycin resistance, they were considered as homozygous and their seeds were harvested to use for following experiments.

### Stress treatments to *Arabidopsis*

Approximately 100 seeds each from the wild-type *Arabidopsis* and transgenic line were sown on MS medium containing 100 mM NaCl, 200 mM NaCl, 200 mM mannitol, 400 mM mannitol, one µM ABA, or two µM ABA to test the germination (radicle emergence). Seeds were firstly incubated for 3 days at 4 °C in darkness to break seed dormancy, and then transferred into a growth incubator keeping 23 °C. The germination was scored daily for 9 days after transferring to 23 °C. For plant growth assays, seeds of wild-type *Arabidopsis* and transgenic lines were sown on MS medium to germinate (radicle emergence). The germinated seeds were then transferred to MS medium containing 100 mM NaCl, 200 mM NaCl, 200 mM mannitol, 400 mM mannitol, one μM ABA, or two μM ABA, and the seedlings were photographed on day 10 after transferring. Three biological replicates were performed.

For expression analysis of previously identified stress-inducible genes, *RD29B* and *RD22*, 3-week-old wild type and transgenic seedlings were sprayed with water (CK), 200 mM NaCl, and 400 mM mannitol to ensure total coverage of the foliage area, respectively. For expression analysis of previously identified ABA-inducible genes, *ABI3*, 3-week-old wild type and transgenic seedlings were also sprayed with water (CK) and 10 µM ABA, respectively. A total of 3 h after stress or ABA treatments, total RNAs of seedlings were then extracted, and qRT-PCR analyses were performed. Three biological replicates were performed.

### Quantitative real-time PCR (qRT-PCR) validation

AMV RNA PCR Kit 3.0 (Takara, Kusatsu, Japan) was used to reverse-transcribe the total RNA according to the manufacturer’s protocol. SYBR Premix Ex Taq™ Kit (Takara, Kusatsu, Japan) was used to perform qRT-PCR reactions with primers for *BjABR1*, *RD29B*, *RD22*, *ABI3*, and *18S* rRNA genes. The *18S* rRNA gene was used as the internal control. The primer pairs are listed in Table S1. A Bio-Rad iQ5 realtime PCR detection was used to perform the qRT-PCR reactions. Three replicates per sample were examined.

### Identification and phylogenetic analysis of *ABR1* homologous genes in *Brassica* species

Five whole-genome sequenced *Brassica* species were used in the present study, including three diploid species (*B. oleracea*, *B. rapa*, and *B. nigra*) and two allotetraploid species (*B. juncea* and *B. napus*). The genomic information of *B. rapa* (version 2.0), *B. nigra* (version 1.1), *B. juncea* (version 1.1), and *B. napus* (version 5) was provided in the *Brassica* Database (BRDB, http://brassicadb.org/brad/index.php), while the genomic information of *B. oleracea* (version 2.1) was provided in the Ensembl Plants Database (http://plants.ensembl.org/Brassica_oleracea/Info/Index). The genomic information of *A. thaliana* was also provided in BRDB. The cloned protein sequence, intron sequence, and promoter sequence of *BjABR1* gene was analyzed by BLAST searches against *B. juncea* database of BRDB. The amino acid sequence of *AtABR1* (*AT5g64750*) gene was analyzed by BLAST searches against *A. thaliana* database of BRDB to confirm no other *ABR1* homologous genes in *A. thaliana*. The amino acid sequence of *AtABR1* gene was submitted as a query to identify *ABR1* homologous genes in *Brassica* species, including *B. nigra*, *B. rapa*, *B. oleracea*, *B. juncea*, and *B. napus*, and the genes whose sequence similarity scores were greater than 300 were *ABR1* homologous genes, while the other genes, whose sequence similarity scores were all less than 100 were not homologous to *ABR1* gene.

The nucleotide or amino acid sequences of the *ABR1* homologous genes were aligned using the software DANMAN 7.0 to view their nucleotide or amino acid sequences changes. Multiple alignment of amino acid sequences of the *ABR1* homologous genes were performed together using ClustalX (Thompson, Higgins & Gibson, 1994) with default options and a nj format file containing genetic distance was generated. One minus the genetic distance was the similarity of pairwise sequences. A subsequent manual alignment correction was accomplished by using MEGA 5.1 (Tamura et al., 2011). Phylogenetic trees were constructed by means of the bootstrap neighbor-joining (NJ) method and a Kimura 2-parameter model that were provided by MEGA 5.05. The stability of internal nodes was assessed by bootstrap analysis with 1,000 replicates. The physical location data of exons and introns of *ABR1* homologous genes were retrieved from the genomic information and the exon-intron structures were displayed by using the Gene Structure Display Server (GSDS, http://gsds.cbi.pku.edu.cn).

### Semi-quantitative analysis of *ABR1* homologous genes in tuber mustard

The spatial and temporal expression of six *ABR1* homologous genes of tuber mustard, *BjuA008532*, *BjuA008665*, *BjuA023050*, *BjuA032667*, *BjuB001787*, and *BjuB007684_correction*, were examined using semi-quantitative method. AMV RNA PCR Kit 3.0 (Takara, Kusatsu, Japan) was used to reverse-transcribe the total RNAs of roots, stems, and leaf of 14-week-old and 16-week-old tuber mustard, respectively. To control for genomic DNA contamination, a reaction lacking reverse transcriptase was performed in parallel for each sample. PCR amplification of a 235 bp fragment of tuber mustard *Actin3* gene (*BjuA014894*), using intron-flanking and gene-specific primers (Table S1), was controlled for the presence of equal amounts of cDNA template in each reaction. Gene-specific primers of *BjuA008532*, *BjuA008665*, *BjuA023050*, *BjuA032667*, *BjuB001787*, and *BjuB007684_correction* genes were designed according to specific region of nucleotide sequences based on the alignment result of nucleotide sequences among the *ABR1* homologous genes. These gene-specific primers were listed in Table S1 and used to amplify the coding regions of *BjuA008532* (286 bp), *BjuA008665* (303 bp), *BjuA023050* (324 bp), *BjuA032667* (371 bp), *BjuB001787* (228 bp), and *BjuB007684_correction* (417 bp) by PCR. The amplified products were also validated by sequencing. In the PCR reactions of six *ABR1* homologous genes, three parameters, the annealing temperature, the extension time, and the quantities of templates were set according to Tm values of primers, the length of amplified segments, and equal amounts of cDNA template in each reaction adjusted by *Actin3* gene, respectively, and the cycle numbers all are 30 times.

## Results

### Cloning and sequence analysis of *BjABR1* promoter

**Figure 1:**
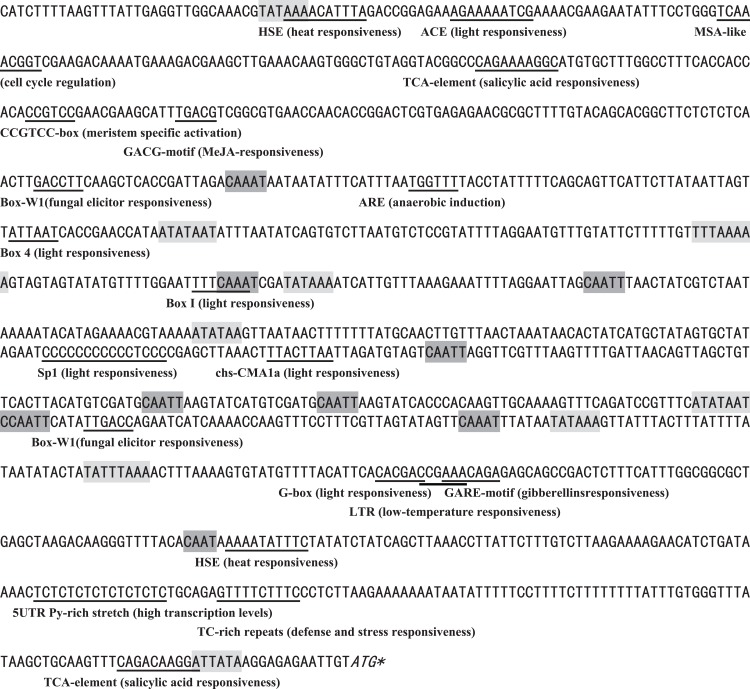
Analysis of the *BjABR1* promoter sequence. The translation start codon was indicated by italics and an asterisk. The putative TATA-box and CAAT-box are indicated with light shadow and dark shadow, respectively, and the other cis-acting elements were indicated by underlining.

With reference to the genomic DNA sequence of *BjABR1*, a 1,212 bp sequence upstream of the coding region was isolated using the genome walking method. A promoter motif search was performed using the PLANTCARE database showed that a number of putative plant cis-elements were present. As shown in Fig. 1, some hormone-related elements were recognized, including the MeJA-responsive element (TGACG-motif), salicylic acid responsive element (TCA-element), and gibberellin-responsive element (GARE-motif); some stress-related elements were recognized, such as heat stress responsiveness element (HSE), low-temperature responsiveness element (LTR), defense and stress responsiveness element (TC-rich repeats), fungal elicitor responsive element (Box-W1), anaerobic induction element (ARE); light-responsive elements, such as the Box 4, ACE, Box I, G-box, Sp1, chs-CMA1a were observed; some development-related elements were also explored, including meristem specific activation element (CCGTCC-box), and cell cycle regulation element (MSA-like). The presence of these putative cis-acting elements indicates that the *BjABR1* gene could function in hormone- and stress-related responses, and plant development.

### Expression patterns of *BjABR1* in response to ABA and abiotic stress in tuber mustard

**Figure 2:**
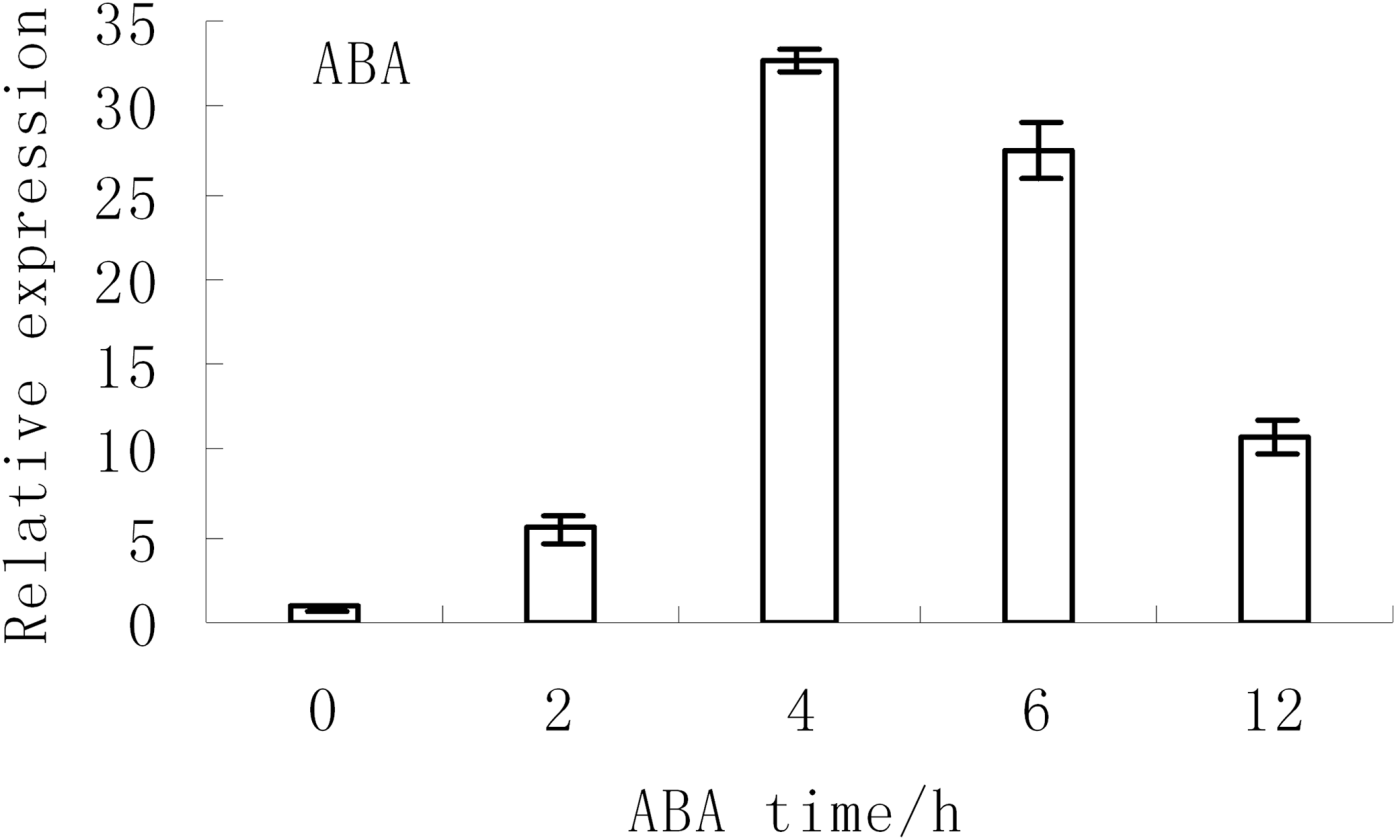
Expression analysis of *BjABR1* in tuber mustard under ABA condition. One-month-old seedlings were treated with 10 μM ABA. Total RNAs were extracted, and qRT-PCR analyses were performed. Error bars represent SE for three independent experiments.

The expression levels of *BjABR1* under abiotic stress conditions were investigated in tuber mustard in a previous study. The expression of *BjABR1* were strongly induced at 2 and 6 h after NaCl and Mannitol treatment, respectively, then decreased, and the expression of *BjABR1* increased gradually with time under cold stress (Xiang et al., 2014). The expression of *BjABR1* was also inducible by ABA. As shown in Fig. 2, the transcripts of *BjABR1* increased rapidly and accumulated to the peak at 4 h after ABA treatment, then declined gradually. It is indicated that *BjABR1* gene may function in responses to ABA and abiotic stress in tuber mustard.

### Alleviative plant sensibility to ABA and abiotic stress in transgenic *Arabidopsis* compared to wild type

**Figure 3:**
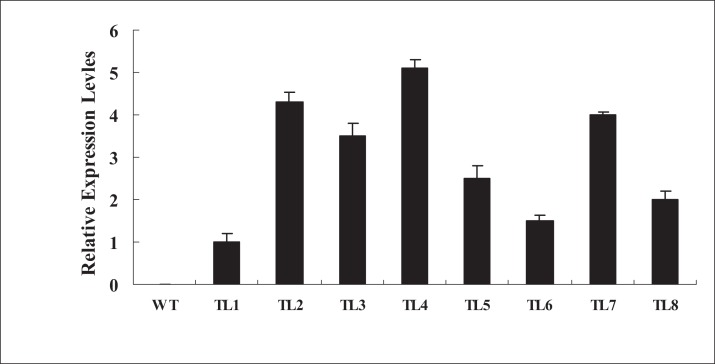
qRT-PCR analysis of *BjABR1* expressing in wild-type *Arabidopsis* and transgenic lines. Total RNAs were extracted from different transgenic lines of T3 generation and wild type plants for qRT-PCR analysis. WT, wild type; TL1, TL2, TL3, TL4, TL5, TL6, TL7, and TL8, transgenic line 1, 2, 3, 4, 5, 6, 7, and 8. Error bars represent SE for three independent experiments.

To further investigate the biological functions of *BjABR1* gene, the transgenic *Arabidopsis* plants expressing *BjABR1* gene were generated. The expression levels of *BjABR1* in transgenic lines were examined using qRT-PCR. All transgenic lines constitutively expressed higher levels of *BjABR1* transcript compared to the wild-type plants (Fig. 3), and three transgenic lines expressing more *BjABR1* transcripts, TL2, TL4, and TL7, were used to the following study.

**Figure 4:**
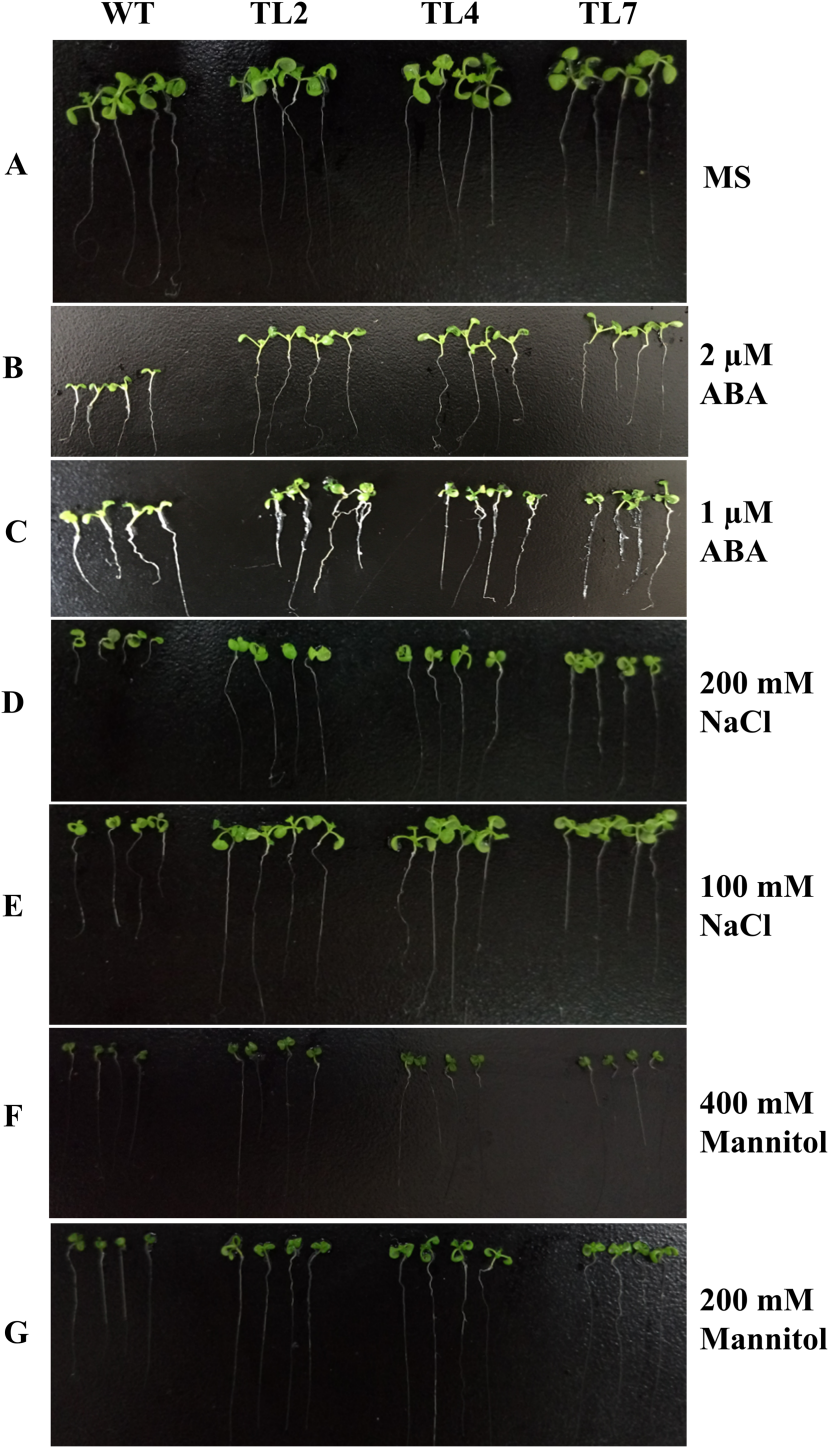
Seedlings growth of wild type and transgenic lines on MS with different levels of ABA, NaCl, or Mannitol. (A) Seedlings growth on MS. (B–G) Seedlings growth on MS with two μM ABA, one μM ABA, 200 mM NaCl, 100 mM NaCl, 400 mM Mannitol, and 200 mM Mannitol, respectively. The photograph was taken on day 10 after germinating.

**Figure 5:**
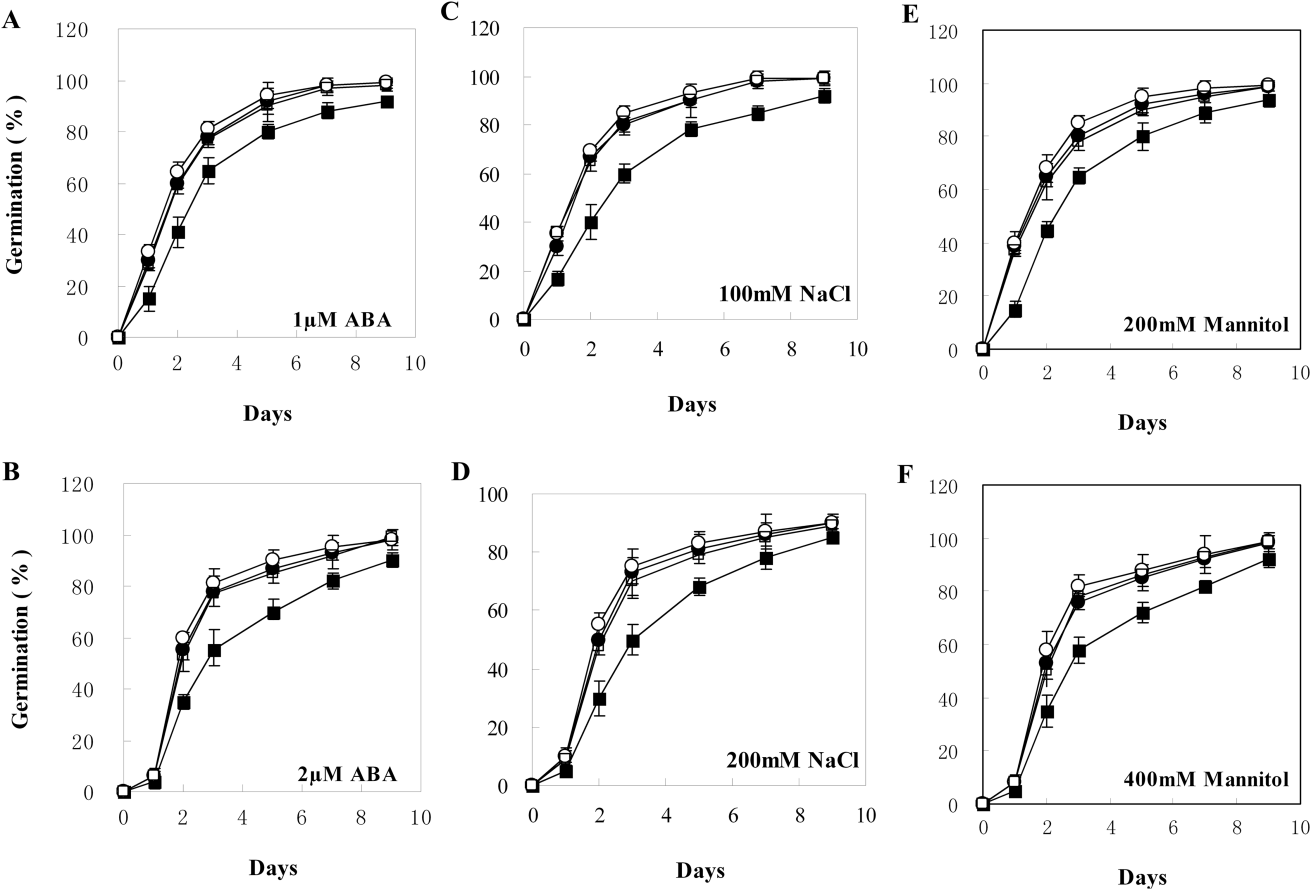
Seed germination of wild type and transgenic lines on MS with different levels of ABA, NaCl, or Mannitol. (A–F) Seed germination on MS with one μM ABA, two μM ABA, 100 mM NaCl, 200 mM NaCl, 200 mM Mannitol, and 400 mM Mannitol, respectively. The black squares, black circles, and white circles, and white squares represented wild type, TL2, and TL4, and TL7 transgenic line, respectively. Data represent the mean values with standard errors of three replicates.

The transgenic plants under normal MS medium were indistinguishable from the wild-type *Arabidopsis* (Fig. 4), but they exhibited alleviative plant sensibility to ABA, osmotic stress, and salt stress compared to the wild type (Figs. 4 and 5). Firstly, the seed germination rates of transgenic lines were improved in different levels of ABA and stress conditions (Fig. 5). For example, at one µM ABA, there are about 60% of transgenic seeds germinating and about 40% of wild-type seeds germinating in the second day, and there are more than 90% of transgenic seeds germinating and less than 80% of wild-type seeds germinating in the fifth day. At two µM ABA, there are more than 50% of transgenic seeds germinating and less than 40% of wild-type seeds germinating in the second day, and there are more than 80% of transgenic seeds germinating and less than 70% of wild-type seeds germinating in the fifth day. At 100 mM NaCl, there are more than 60% of transgenic seeds germinating and less than 40% of wild-type seeds germinating in the second day, and there are about 90% of transgenic seeds germinating and about 70% of wild-type seeds germinating in the fifth day. At 200 mM NaCl, there are about 50% of transgenic seeds germinating and about 30% of wild-type seeds germinating in the second day, and there are about 80% of transgenic seeds germinating and less than 70% of wild-type seeds germinating in the fifth day. Similarly, the germination rate of transgenic seeds was greater than that of wild-type seeds on the MS medium with 200 mM or 400 mM mannitol. Secondly, the growth of transgenic seedlings on ABA and stress conditions was comparable to the wild type, showing that the wild-type plants were significantly more inhibited by ABA, osmotic and salt stress compared to the transgenic plants, and the transgenic plants distinctly had more and larger leaves and longer roots compared to WT under the same condition (Fig. 4). It is indicated that *BjABR1* gene involved in responses to ABA and abiotic stresses.

### Altered expression of stress/ABA-induced genes in transgenic *Arabidopsis*

**Figure 6:**
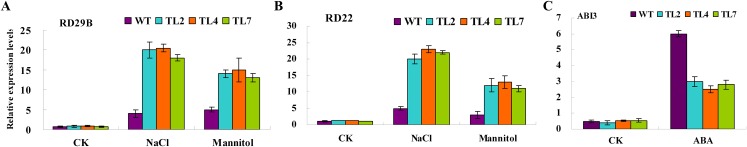
Relative expression levels of stress/ABA-responsive genes in response to stress and ABA treatments in wild-type and transgenic *Arabidopsis*. Three-week-old seedlings were treated with water (CK), 10 μM ABA, 200 mM NaCl, and 400 mM Mannitol, respectively. Total RNAs were then extracted, and qRT-PCR analyses were performed. (A and B) Relative expression levels of stress-responsive genes, *RD29B*, or *RD22*, in response to stress. (C) Relative expression levels of ABA-responsive gene, *ABI3*, in response to ABA. WT, wild type; TL2, TL4, and TL7, transgenic line 2, 4, and 7. Error bars represent SE for three independent experiments.

To elucidate the molecular mechanism of *BjABR1* action during the stress/ABA response, the expression of known stress/ABA-induced genes was analyzed in wild type and transgenic lines following NaCl, mannitol or exogenous ABA application. qRT-PCR analysis was performed to monitor the expression levels of two previously identified stress-inducible genes, *RD29B* and *RD22*, and one ABA-inducible gene, *ABI3*. Under normal growth conditions, transcript levels of *RD29B*, *RD22*, and *ABI3* were comparable in both wild type and transgenic *Arabidopsis* (Fig. 6). The transcript levels of *RD29B* and *RD22*, however, were significantly higher in the transgenic lines compared to the wild type under salt and mannitol conditions (Figs. 6A and 6B), while activity of *ABI3* gene was down-regulated in the transgenic lines (Fig. 6C). The result indicated that alleviative plant sensibility to abiotic stress in transgenic *Arabidopsis* compared to wild type may be due to the increased expression of the stress-induced genes correlating with stress tolerance. The *ABI3* gene product is an important component of ABA signaling pathway (Giraudat et al., 1992) and overexpression of *ABI3* in *Arabidopsis* conferred hypersensitivity to ABA (Lopez-Molina et al., 2002). Therefore, the alleviative plant sensibility of transgenic *Arabidopsis* to ABA may result from the reduced expression of *ABI3* gene in the transgenic *Arabidopsis*.

### Identification and phylogenetic analysis of *ABR1* homologous genes

By BLASTP searches against *B. juncea* database of BRDB using the protein sequence of *BjABR1* gene, the gene, named *BjuA032667* in *B. juncea* database, shared the highest amino acid sequence similarity (about 99%) with *BjABR1* (Fig. S1). The intron, and promoter sequences of *BjABR1* gene cloned by us were also aligned with the corresponding sequences of *BjuA032667* gene, respectively, showing that the intron and promoter sequences between the two genes are also highly similar, and there were only four and five nucleotide changes in their intron and promoter sequences, respectively (Fig. S2). It is believed that *BjABR1* and *BjuA032667* are the same gene and their sequence difference may result from the error of sequencing or PCR amplification, or nucleotide mutation or polymorphism. The *BjuA032667* gene instead of *BjABR1* was used to the following analysis.

By BLASTP searches against *A. thaliana* database of BRDB using the protein sequence of *AtABR1* (*AT5g64750*) gene, the alignment score of *AT5g64750* gene was 571, while the alignment scores of other genes were all less than 100, so there were no genes that were homologous to *AtABR1* except itself in *A. thaliana*.

By BLASTP searches against *B. juncea, B. rapa*, *B. nigra*, and *B. napus* database of BRDB, and *B. oleracea* database in Ensembl Plants using the protein sequence of *AtABR1* gene, three (named *BniB045887-PA*, *BniB004262-PA*, and *BniB047045-PA*), three (named *Scaffold000071.53*_*Bra037794*, *Scaffold000004.823*_*Bra024325*, and *Scaffold000021.116*_*Bra031903*), three (named *Bo9g018330.1*, *Bo3g100930.1*, and *Bo2g166380.1*), six (named *BjuB001787*, *BjuB007684*, *BjuA023050*, *BjuA008665*, *BjuA008532*, and *BjuA032667*), and five (named *GSBRNA2T00079211001*, *GSBRNA2T00000822001*, *GSBRNA2T00047471001*, *GSBRNA2T00086262001*, and *GSBRNA2T00134741001*) *ABR1* homologous genes in *B. nigra*, *B. rapa*, *B. oleracea*, *B. juncea*, and *B. napus* were identified, respectively.

**Figure 7:**
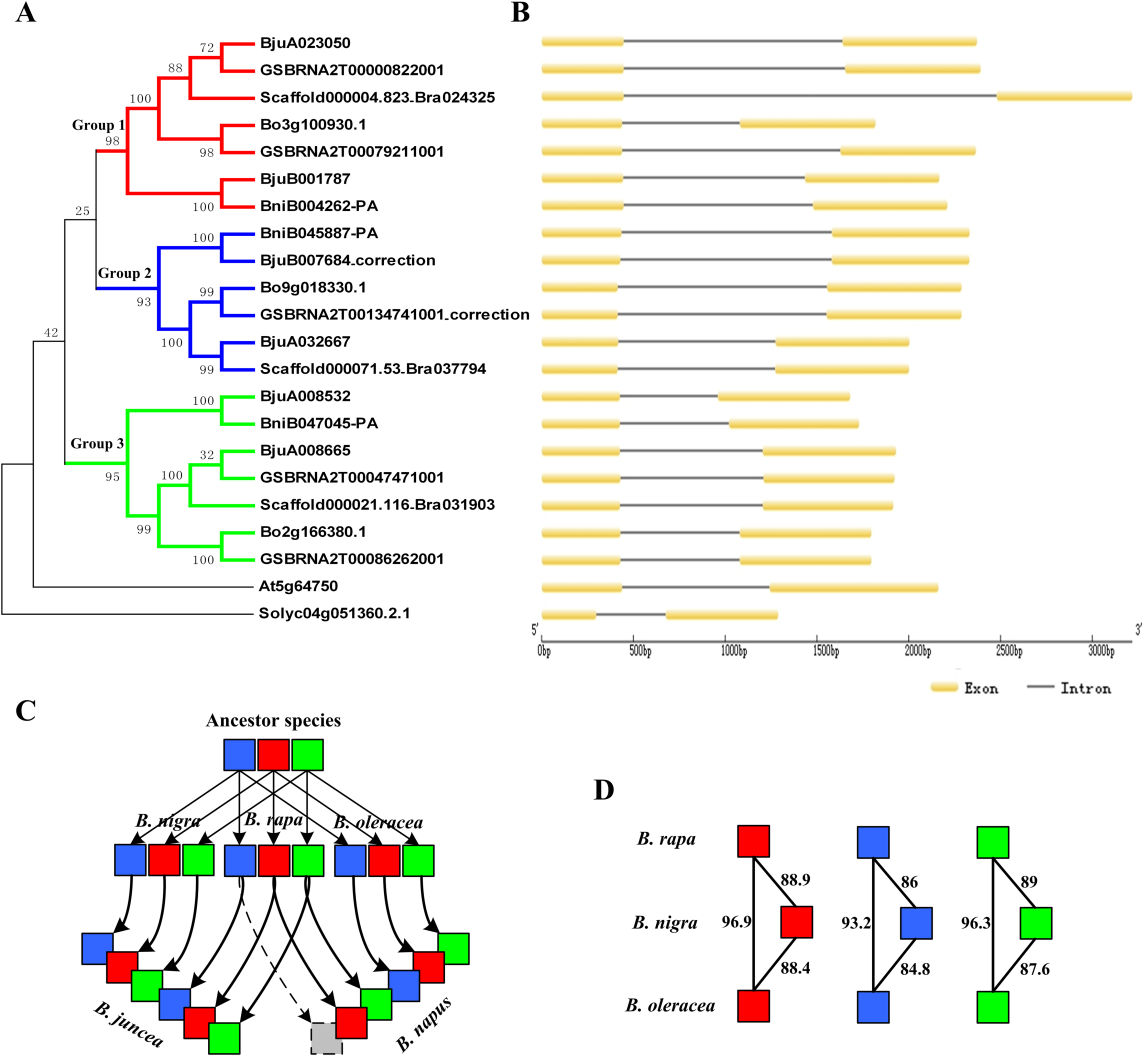
Phylogenetic relationship and gene structure of *ABR1* homologous genes. (A) A phylogenetic tree of 22 *ABR1* homologous genes constructed on the basis of NJ method with a bootstrap value of 1,000 using MEGA5.1. *Solyc04g051360.2.1* gene of *Solanum lycopersicum* was the outgroup gene. The genes in red, blue, and green branches belonged to group 1, 2, and 3, respectively. (B) Exon-intron structure of *ABR1* homologous genes. (C) Evolutionary trajectory of *ABR1* homologous genes in *Brassica* species. Red, blue, and green squares represented the genes in group 1, group 2, and group 3, respectively. The gray square indicated that the gene was lost in *B. napus*. The arrows represented evolutionary trajectory of genes among *Brassica* species. (D) The amino acid sequence similarity percentage among *ABR1* homologous genes of the three diploid *Brassica*. Red, blue, and green squares represented the genes in group 1, group 2, and group 3, respectively. The number near the black line linking two squares represented the sequence similarity percentage between the two genes.

**Figure 8:**
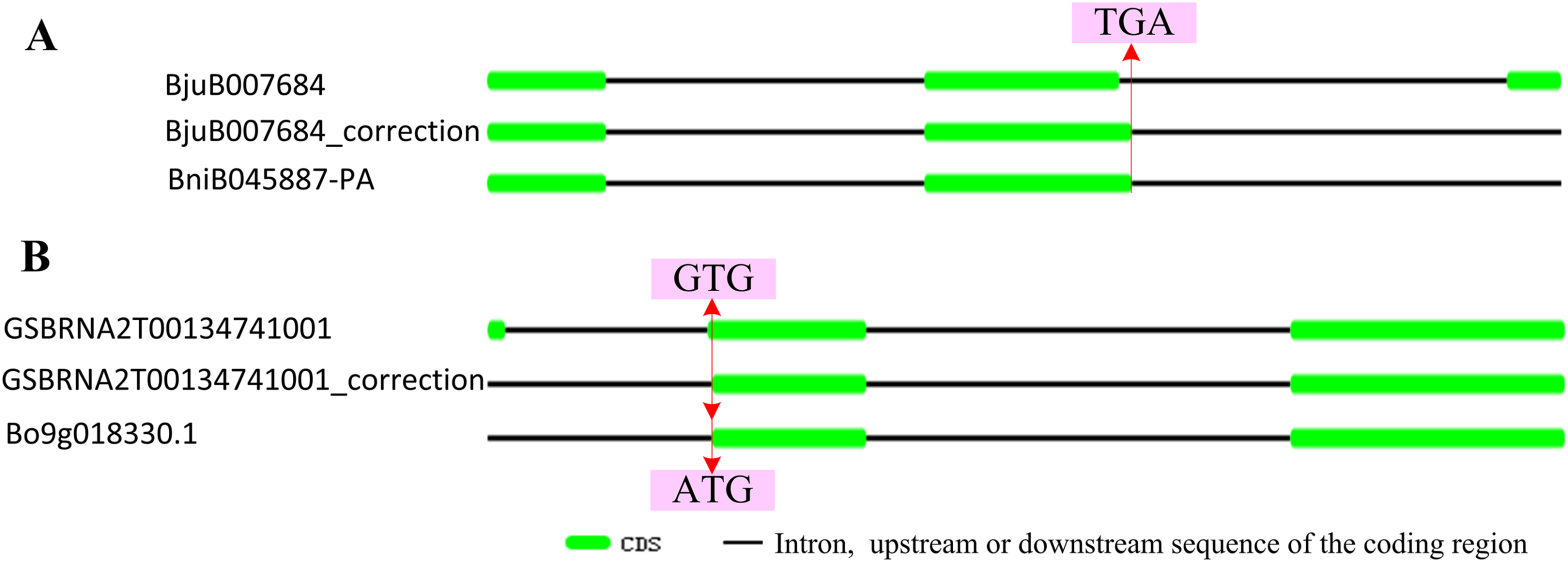
Correctionof exon-intron structure of two *ABR1* homologous genes. (A) Structure of *BjuB007684* and correctedstructure of renamed *BjuB007684*_correction compared to that of *BniB045887-PA*. (B) Structure of *GSBRNA2T00134741001* and correctedstructure of renamed *GSBRNA2T00134741001*_correction compared to that of *Bo9g018330.1*. The red line represented the same location on sequence, and the arrows pointed to the nucleotides in the location.

According to the location data of exons and introns of *ABR1* homologous genes from genomic information, all *ABR1* homologous genes had one intron (Fig. 7B) except *BjuB007684* and *GSBRNA2T00134741001* which had two introns (Fig. 8). Comparing the nucleotide sequence of *BjuB007684* gene with that of its the closest genes, *BniB045887-PA*, the results showed that the first intron sequence, the first and second exon sequence of *BjuB007684* were highly similar with the intron sequence, the first and second exon sequence of *BniB045887-PA*, respectively (Fig. S3), while the second intron and third exon sequence of *BjuB007684* were highly similar with the partial sequence of the second exon and the downstream sequence of the coding region of *BniB045887-PA* gene (Fig. S4). Further sequence alignment analysis showed that the termination codon (TGA) was present in *BjuB007684* in the same location where the termination codon of *BniB045887-PA* in (Fig. 8A), indicating that the second intron and the third exon of *BjuB007684* may be wrongly annotated. Then, the gene structure and the termination codon of *BjuB007684* were corrected as that of *BjuB007684_correction* according to the nucleotide sequence of *BniB045887-PA* (Fig. 8A).

Comparing the nucleotide sequence of *GSBRNA2T00134741001* gene with that of its the closest genes, *Bo9g018330.1*, the results showed that the second intron sequence, the second and third exon sequence of *GSBRNA2T00134741001* were highly similar with the intron sequence, the first and second exon sequence of *Bo9g018330.1*, respectively (Fig. S5), while the first exon sequence and first intron sequence of *GSBRNA2T00134741001* were highly similar with the upstream sequence of the coding region of *Bo9g018330.1* gene (Fig. S6). Further sequence alignment showed that the initiation codon of *Bo9g018330.1* gene is ATG, while the corresponding position of *GSBRNA2T00134741001* gene was GTG (Figs. S5B and S6B), indicating that nucleotide error in *GSBRNA2T00134741001* sequence may be present. Then, the gene structure and the initiation codon of *GSBRNA2T00134741001* were corrected as that of *GSBRNA2T00134741001_correction* according to the nucleotide sequence of *Bo9g018330.1* (Fig. 8B).

**Figure 9:**
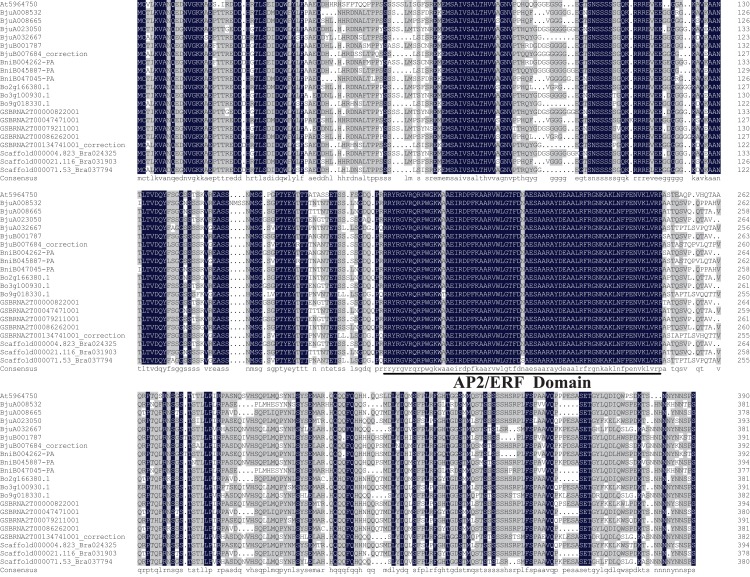
Amino acid sequence alignment of *ABR1* homologous genes of *Brassica* species and *A. thaliana*. Amino acid residues that are conserved in at least eleven of the 21 sequences are shaded, while amino acids identical in all 21 proteins are showed in dark gray. The AP2/ERF domain is underlined.

The amino acid sequences of *ABR1* homologous genes of *Brassica* species and *A. thaliana* were aligned and the results showed that they shared high sequence similarity, especially the AP2/ERF domain (Fig. 9).

The phylogenetic tree was constructed on the basis of NJ method with a bootstrap value of 1,000 using MEGA5.1. The phylogenetic tree revealed that the *ABR1* homologous genes of *Brassica* species were divided into three groups, group1, group 2, and group3, showed, respectively by red, blue, and green branches in Fig. 7A. Each diploid *Brassica* species had three *ABR1* homologous genes (nine genes in total), and the nine genes were divided into three group and each group contained three genes which belonged, respectively to three diploid *Brassica* (Fig. 7A), indicating that the ancestor species of three diploid *Brassica* also had three *ABR1* homologous genes which were all then inherited to each diploid *Brassica*. The genes of *B. rapa* and *B. oleracea*, *Bo3g100930.1* and *Scaffold000004.823*_*Bra024325*, *Bo9g018330.1* and *Scaffold000071.53*_*Bra037794*, and *Bo2g166380.1* and *Scaffold000021.116*_*Bra031903* shared closer relationship compared to *BniB004262-PA*, *BniB045887-PA*, and *BniB047045-PA* of *B. nigra*, respectively (Fig. 7A), and the amino acid sequence similarity percentage of *ABR1* homologous genes between *B. rapa* and *B. oleracea* were much greater than that between *B. rapa* and *B. nigra*, and between *B. oleracea* and *B. nigra* (Fig. 7D), indicating that the *ABR1* homologous genes may have experienced more rapid evolution in *B. nigra* species.

The two allotetraploid *Brassica* species, *B. juncea* and *B. napus*, have six and five *ABR1* homologous genes, respectively. *B. juncea* was originated by hybridization between diploid *B. rapa* and diploid *B. nigra*, while *B. napus* was originated by hybridization between diploid *B. rapa* and diploid *B. oleracea*. The phylogenetic tree revealed that *B. juncea* inherited all the six *ABR1* homologous genes from *B. rapa* and *B. nigra* with no gene lost, while *B. napus* inherited all the three *ABR1* homologous genes from *B. oleracea* and two *ABR1* homologous genes from *B. rapa* with one gene (*Scaffold000071.53*_*Bra037794*) lost (Fig. 7A). Furthermore, the intron sequences, and upstream and downstream sequence of the coding sequence of the *ABR1* homologous genes in the same group were analyzed by pairwise sequence alignment (data not shown) and the results of sequence similarity among these genes were consistent with the gene relationships shown in the phylogenetic tree. Therefore, the evolutionary trajectory of *ABR1* homologous genes in *Brassica* species can be summarized that the ancestor of *B. rapa*, *B. nigra*, and *B. oleracea* had three *ABR1* homologous genes, and *B. rapa*, *B. nigra*, and *B. oleracea* inherited all the three *ABR1* homologous genes from the ancestor species, respectively, and then, *B. juncea* inherited all the six *ABR1* homologous genes from *B. rapa* and *B. nigra* with no gene lost, while *B. napus* inherited all three *ABR1* homologous genes from *B. oleracea* and two *ABR1* homologous genes from *B. rapa* with one gene lost (Fig. 7C).

### Expression analysis of *ABR1* homologous genes in tuber mustard

**Figure 10:**
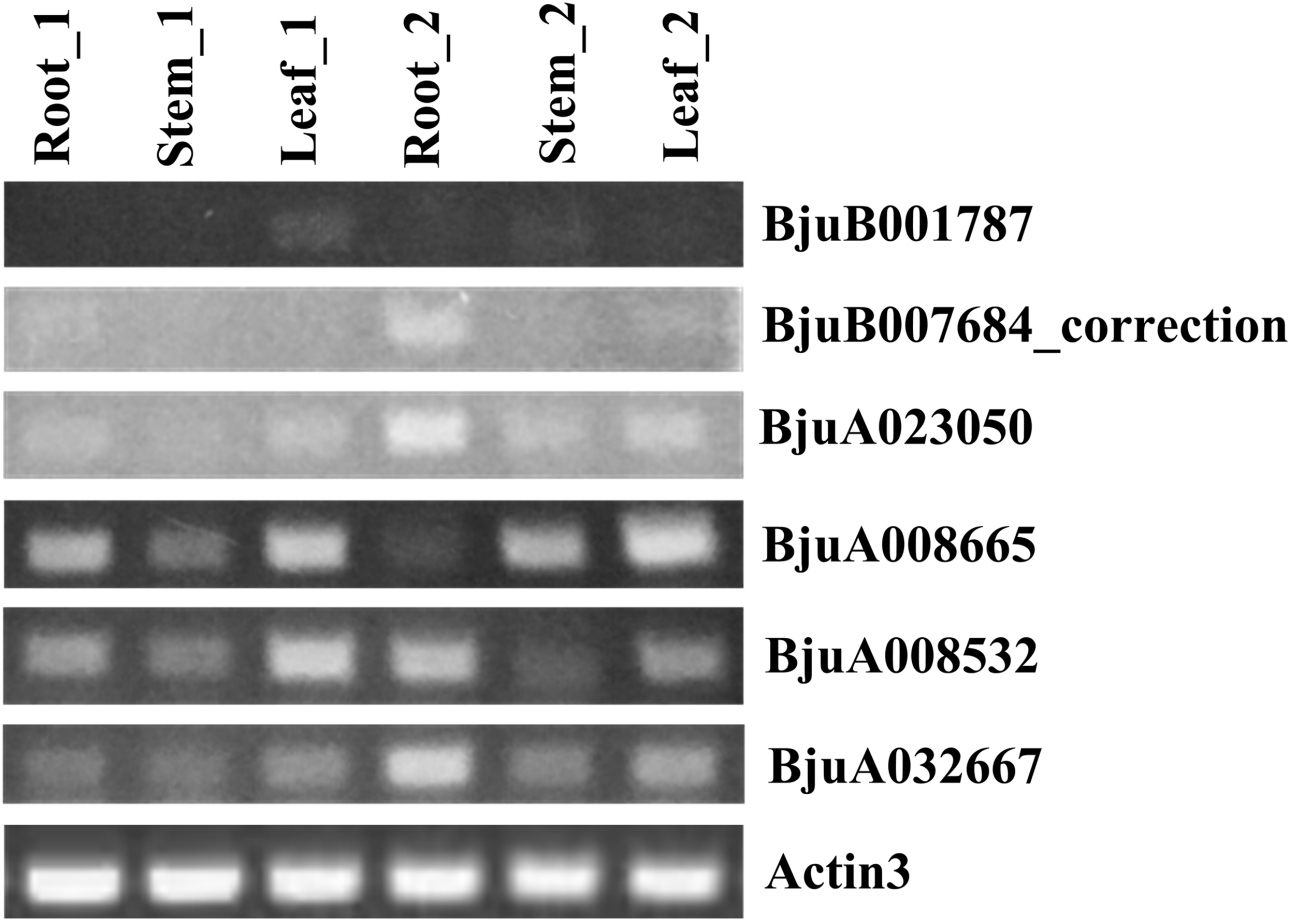
Expression analysis of six *ABR1* homologous genes of tuber mustard. Root_1, Stem_1, and Leaf_1 represented roots, stems, and leaves before the start of stem swelling, respectively. Root_2, Stem_2, and Leaf_2 represented roots, stems, and leaves in the stem swelling stage, respectively.

To understand the roles of six *ABR1* homologous genes, *BjuA008532*, *BjuA008665*, *BjuA023050*, *BjuA032667*, *BjuB001787*, and *BjuB007684_correction*, in growth and development of tuber mustard, their spatial and temporal expression in roots, stems, leaves before and after the start of stem swelling were examined using semi-quantitative method, respectively. The analysis revealed that the six *ABR1* homologous genes possessed different expression models (Fig. 10). *BjuA008665*, *BjuA008532*, *BjuA032667* were more clearly expressed in samples compared to *BjuB001787*, *BjuA023050*, and *BjuB007684_correction*, while the expression levels of each gene in six samples were different (Fig. 10). For example, *BjuA032667* was expressed in all samples, especially in the roots in stem swelling stage (Fig. 10). It is indicated that the *ABR1* homologous genes may play a role in different tissues in different growing stages.

## Discussion

Many AP2/ERF superfamily genes were characterized to play an important role in the regulation of different development processes and stress responses in plants. In our previous study, we have identified 1,042 differentially expressed genes in stem development in tuber mustard by RNA sequencing, and the expression of Unigene 140028_num2_yongan (Sun et al., 2012) known as *BjABR1* gene here between un-swollen stem and swollen stem was significantly different (Sun et al., 2012). We speculated that *BjABR1* gene may play an important role in plant growth. Therefore, the full-length cDNA of *BjABR1* was cloned later (Xiang et al., 2014), and the function of *BjABR1* was further studied in this study.

The protein sequence of *BjABR1* gene cloned from tuber mustard was predicted to contain a conserved AP2/ERF domain and it was located in nucleus by onion epidermal subcellular localization analysis, indicating that *BjABR1* gene was a potential AP2/ERF transcription factor gene (Xiang et al., 2014). *BjABR1* gene hared high amino acid sequence similarity (about 77%) with *AtABR1* gene of *A. thaliana*. The *AtABR1* gene of *A. thaliana* responses to ABA and stress conditions including cold, high salt, and drought, and functions as a repressor of ABA response in *Arabidopsis* (Pandey et al., 2005). The promoter sequence analysis of *BjABR1* gene showed that there were many hormone and stress related cis-acting elements, indicating that *BjABR1* may function in hormone and stress responses. Then, the expression analysis of *BjABR1* gene showed that it was induced under ABA and stress conditions including cold, high salt, osmotic stress in tuber mustard, and overexpression of *BjABR1* gene in *Arabidopsis* can alleviate plant sensitivity to ABA and abiotic stress, indicating that *BjABR1* gene is involved in ABA and abiotic stress signaling. The result was also consistent with hypersensitivity of the *abr1* mutant *Arabidopsis* to ABA and osmotic stress (Pandey et al., 2005), indicating that the function of *BjABR1* gene of tuber mustard was similar with that of *AtABR1* gene of *Arabidopsis*, playing a role in responses to ABA as a repressor and abiotic stress. The expression analysis of known stress/ABA-induced genes between wild type and transgenic *Arabidopsis* under stress/ABA conditions indicated the transcript levels of stress-inducible genes, *RD29B* and *RD22*, were improved in transgenic lines, while the expression of the ABA-inducible gene, *ABI3*, was reduced, indicating that the alleviative plant sensibility of transgenic *Arabidopsis* to abiotic stress may be due to the increased expression of the stress-induced genes, while the alleviative plant sensibility of transgenic *Arabidopsis* to ABA may result from the reduced expression of ABA-induced genes.

The genomes of *B. juncea* have been published recently (Yang et al., 2016). By BLAST search and sequence alignment, the amino acid or nucleotide sequences of *BjABR1* gene cloned by us were not the same with its the closed gene *BjuA032667* in the *B. juncea* database, but their sequences were highly similar. Therefore, it is believed that *BjABR1* and *BjuA032667*are the same gene, whose sequence difference may result from the error of sequencing or PCR amplification, or nucleotide mutation or polymorphism.

*B. juncea* and *B. napus* were formed by hybridization between *B. rapa* and *B. nigra*, and between *B. rapa* and *B. oleracea*, respectively. With the availability of genomic data of *B. rapa*, *B. oleracea*, *B. nigra*, *B. napus*, and *B. juncea* (Wang et al., 2011b; Liu et al., 2014; Parkin et al., 2014; Chalhoub et al., 2014; Yang et al., 2016), we can analyze the evolution of *ABR1* homologous genes in the five *Brassica* species. Our results found that the *B. rapa*, *B. nigra*, and *B. oleracea* inherited all three *ABR1* homologous genes from their ancestor species, respectively, and then, *B. juncea* inherited all the six *ABR1* homologous genes from *B. rapa* and *B. nigra* with no gene lost, while *B. napus* inherited all the three *ABR1* homologous genes from *B. oleracea* and two *ABR1* homologous genes from *B. rapa* with one gene lost, indicating that only a few genes was lost in the evolution of *Brassica* species. Song et al. (2016) also revealed that *B. napus* has 515 *AP2/ERF* genes, about fewer than twice of those in *B. rapa* (281) or *B. oleracea* (281), showing likely about 9% gene losses in each subgenome after hybridization. However, the mechanism of gene loss in *Brassica* species is not clear and needs to be further studied.

The *BjABR1* gene of tuber mustard has the similar function in responses to hormone and abiotic stress with *AtABR1* gene of *Arabidopsis*. The evolution analysis showed that the other *ABR1* homologous genes of *Brassica* species had closer relationships with *BjABR1* gene compared to *AtABR1*, indicating that these *ABR1* homologous genes may also have the same function in responses to hormone and abiotic stresses.

In this study, we also found that *ABR1* homologous genes between *B. rapa* and *B. oleracea* shared higher sequence similarity and closer evolution relationship compared to that of *B. nigra*, indicating that the *ABR1* homologous genes had experienced more rapid evolution in *B. nigra* species. We speculate that *B. rapa* and *B. oleracea* shared closely-related evolution relationship compared to *B. nigra* in the three diploid *Brassica* species. However, it also needs to be further studied. Moreover, the spatial and temporal expression analysis showed that six ABR1 homologous genes of tuber mustard possessed different expression model, indicating that the *ABR1* homologous genes may play a role in different tissues in different growing stages.

Our study exhibits firstly the evolutionary relationships of a class of homologous genes in *Brassica* species. Our study will help to reveal and understand the mechanisms of *Brassica* species evolution and the roles of homologous genes in *Brassica* species.

## Conclusions

*BjABR1*, an AP2/ERF superfamily gene, from tuber mustard (*B. juncea* var. *tumida* Tsen et Lee), functioned in phytohormone and abiotic stress responses. Especially, *BjABR1* gene can alleviate plant sensitivity to ABA and osmotic stresses, probably due to changes in stress/ABA-induced genes expression. *ABR1* homologous genes were identified in *Brassica* species and they shared high sequence similarity. From the ancestor of diploid *Brassica* species to allotetraploids, *ABR1* homologous genes were all inherited except a loss in *B. napus*, indicating that *ABR1* homologous genes possessed greater hereditary conservation in *Brassica* species. *B. rapa* and *B. oleracea* may share closer relationship among the three diploid species. Moreover, the six *ABR1* homologous genes of tuber mustard possessed different spatio-temporal expression models. These results imply that the *ABR1* homologous genes in *Brassica* species may possess similar function in ABA and abiotic stress responses, but play a role in different tissues and growing stages of plant. The study will provide the foundation to the functional research of *ABR1* homologous genes in the *Brassica* species and help to reveal and understand the evolution mechanisms of *Brassica* species.

## Supplemental Information

10.7717/peerj.6071/supp-1 Supplemental Information 1 Protein-coding sequence alignment between *BjuA032667* gene and *BjABR1* gene. Amino acid conserved in two sequences are showed in dark gray.

10.7717/peerj.6071/supp-2 Supplemental Information 2 Comparison of the intron and promoter (about 1,200 bp) sequence of *BjABR1* and *BjuA032667*. (A) Intron sequence alignment between *BjABR1*and *BjuA032667*. (B) Promoter sequence alignment between *BjABR1* and *BjuA032667*. Nucleotides conserved in two sequences are showed in dark gray.

10.7717/peerj.6071/supp-3 Supplemental Information 3 Comparison of the intron and exon sequence of *BjuB007684* and *BniB045887-PA*. (A) Sequence alignment between the intron of *BniB045887-PA* and the first intron of *BjuB007684*. (B) Sequence alignment between the first exon of *BniB045887-PA* and the first exon of *BjuB007684*. (C) Sequence alignment between the second exon of *BniB045887-PA* and the second exon of *BjuB007684*. Nucleotides conserved in two sequences are showed in dark gray.

10.7717/peerj.6071/supp-4 Supplemental Information 4 Raw data for gene expression.

10.7717/peerj.6071/supp-5 Supplemental Information 5 Sequences of primers used for qRT-PCR and semi-quantitative analysis. F, Forward primer; R, reverse primer.

10.7717/peerj.6071/supp-6 Supplemental Information 6 Raw data for germination.

10.7717/peerj.6071/supp-7 Supplemental Information 7 Comparison of the second intron sequence and third exon sequence of *BjuB007684* with the partial sequence of the second exon and the downstream sequence of the coding region of *BniB045887-PA* gene. The partial sequence of the second exon of *BniB045887-PA* gene was signed by deep pink box and the rest of sequence was its downstream sequence of the coding region, while the third exon sequence of *BjuB007684* was signed by red underline and the rest of sequence was its second intron sequence. Nucleotides conserved in two sequences are showed in dark gray.

10.7717/peerj.6071/supp-8 Supplemental Information 8 Full-length gels and blots.

10.7717/peerj.6071/supp-9 Supplemental Information 9 Comparison of the intron and exon sequence of *GSBRNA2T00134741001* and *Bo9g018330.1*. (A) Sequence alignment between the intron of *Bo9g018330.1* and the second intron of *GSBRNA2T00134741001*. (B) Sequence alignment between the first exon of *Bo9g018330.1* and the second exon of *GSBRNA2T00134741001*. (C) the second exon of *Bo9g018330.1* and the third exon of *GSBRNA2T00134741001*. Nucleotides conserved in two sequences are showed in dark gray.

10.7717/peerj.6071/supp-10 Supplemental Information 10 Comparison of the first exon sequence and first intron sequence of *GSBRNA2T00134741001* with the upstream sequence of the coding region of *Bo9g018330.1* gene. The first exon sequence of *GSBRNA2T00134741001* gene was signed by red box. Nucleotides conserved in two sequences are showed in dark gray.

## Additional Information and Declarations

### Data Availability

The following information was supplied regarding data availability: The raw measurements are provided in the Supplemental Information.
