# Supplementary material for: A tuber mustard AP2/ERF transcription factor gene, BjABR1, functioning in abscisic acid and abiotic stress responses, and evolutionary trajectory of the ABR1 homologous genes in Brassica species
Source: PeerJ. 2018 Dec 11;6:e6071. doi: 10.7717/peerj.6071 (PMC6294115; doi:10.7717/peerj.6071)
Supplement: Supplemental Information 1 — Amino acid conserved in two sequences are showed in dark gray. [file peerj-06-6071-s001.pdf]

```
BjuA032667    MCALKVANQEGNVGKKSEPTTREDDDHRTLSDIDQWLYSFSAEDDYLLHRDNSLTPPSSSLSISREKEMSAIVSALTHVVAGNVPTRQYGGEGTSNSSSS    100
BjABR1        MRALKVANQEGNVGKKAEPTTREDDDHRTLSDIDRWLYSFSAEDDY.LHRDNSLTPPSSSLSISREKEMSAIVSALTHVVAGNVPTRQYGGEGTSNSSSS     99
Consensus     mcalkvanqegnvgkkaepttredddhrtlsdidqwlysfsaeddyllhrdnsltppssslsisrekemsaivsalthvvagnvptrqyggegtsnssss

BjuA032667    SGQKRRRELEEGGGGGKDVKAANTLTVDQYFAGGSSSSRVGEASSNMSGSVPTYEYTTTPNTNTETSLLSGDGPQRRYRGVRQRPWGKWAAEIRDPFKAA    200
BjABR1        SGQKRRRELEEGGGGGKDVKAANTLTVDQYFAGGSSSSRVGEASSNMSGSVPTYEYTTTPNANTETSLLSGDGPQRRYRGVRQRPWGKWAAEIRDPFKAA    199
Consensus     sgqkrrreleegggggkdvkaantltvdqyfaggssssrvgeassnmsgsvptyeytttpnantetsllsgdgpqrryrgvrqrpwgkwaaeirdpfkaa

BjuA032667    RVWLGTFDNAESAARAYDEAALRFRGNKAKLNFPENVKLVRPASTTPTLSVPQTAVQRPTQLRNSGSTSTILPVRHASDQNVHSQLLMQSYNFSYLELAH    300
BjABR1        RVWLGTFDNAESAARAYDEAALRFRGNKAKLNFPENVKLVRPASTTPTLSVPQTAVQRPTQLRNSGSTSTILPVRHASDQNVHSQLLMQSYNFSYLELAH    299
Consensus     rvwlgtfdnaesaaraydeaalrfrgnkaklnfpenvklvrpasttptlsvpqtavqrptqlrnsgststilpvrhasdqnvhsqllmqsynfsylelah

BjuA032667    HQQQFQHQQQSLYDQVSFPLRFGHTGGSTMQSTSTSLSRSMFSPAAVQPKPESASETGHLFDLQSLGKASNNNNNYNNSPS                       381
BjABR1        HQQQFQHQQQSLYDQVSFPLRFGHTGGSTMQSTSTSLSRSMFSPAAVQPKPESASETGHLFDLQSLGKASNNNNNYNNSPS                       380
Consensus     hqqqfqhqqqslydqvsfplrfghtggstmqststslsrsmfspaavqpkpesasetghlfdlqslgkasnnnnnynnsps
```
