# Supplementary material for: A tuber mustard AP2/ERF transcription factor gene, BjABR1, functioning in abscisic acid and abiotic stress responses, and evolutionary trajectory of the ABR1 homologous genes in Brassica species
Source: PeerJ. 2018 Dec 11;6:e6071. doi: 10.7717/peerj.6071 (PMC6294115; doi:10.7717/peerj.6071)
Supplement: Supplemental Information 2 — (A) Intron sequence alignment between BjABR1and BjuA032667. (B) Promoter sequence alignment between BjABR1 and BjuA032667. Nucleotides conserved in two sequences are showed in dark gray. [file peerj-06-6071-s002.pdf]

A

```
BjuA032667   GTATTTTTTCATTTTTTTTAATAATAACACACGCGTGGACAATCAGCTTAAGTTTGAATTATCTCACGTGTATGTATGTGTATATAAAAGAATGGTGGAT   100
BjABR1       GTATTTTTTCAGTTTTTTTAATAATAACACACGCGTGGACAATCAGCTTAAGTTTGAATTATCTCACGTGTATGTATGTGTATATAAAAGAATGGTGGAT   100
Consensus    gtattttttcagtttttttaataataacacacgcgtggacaatcagcttaagtttgaattatctcacgtgtatgtatgtgtatataaaagaatggtggat

BjuA032667   TCTCAAAACACAAAAACTATTTTTCCTTCAATTTTGTCAAAACTTCAAATGAATCTTGAGATTTTGAAAACATTTATGATAAATGTTTTAATAAATTTGT   200
BjABR1       TCTCAAAACACAAAAACTATTTTTCCTTCAATTTTGTCAAAACTTCAAATGAATCTTGAGATTTTGAAAACATTTATGATAAATGTTTAAATAAATTTGT   200
Consensus    tctcaaaacacaaaaactatttttccttcaattttgtcaaaacttcaaatgaatcttgagattttgaaaacatttatgataaatgtttaaataaatttgt

BjuA032667   GATGTGGTATATTATTATATACATCAAGTGTATGAGTCACATCATATAAAACACAAAATCTAAATATTTGCTCTTTTGTTGTGTTTATTTGTTTAATTTT   300
BjABR1       GATGTGGTATATTATTATATACATCAAGTGTATGAGTCACATCATATAAAACACAAAATCTAAATATTTGCTCTTTTGTTGTGTTTATTTGTTTAATTTT   300
Consensus    gatgtggtatattattatatacatcaagtgtatgagtcacatcatataaaacacaaaatctaaatatttgctcttttgttgtgtttatttgtttaatttt

BjuA032667   AATGAAAAAAGTTGCTCTTTTTTTTTGCACTCACGCAATATACAAAATTAACCGCAATTTTTTTTTGCTTTCCTCTAGAAATTCCATGAGTTCTTTTCAT   400
BjABR1       AATGAAAAAAGTTGCTCTTTTTTTTTGCACTCACGCAATATACAAAATTAACCGCAATTTTTTTTTGCTTTCCTCTAGAAATTCCATGAGTTCTTTTCAT   400
Consensus    aatgaaaaaagttgctctttttttttgcactcacgcaatatacaaaattaaccgcaatttttttttgctttcctctagaaattccatgagttcttttcat

BjuA032667   AAATGAGTAGTTTTTTTTCCACAGGACTATCGAAGAACCAATGCATATACCAGTTTCCAAGTGTTTTTTTTTTGAAAAAATGGTTTAATATTTCTTAAAT   500
BjABR1       AAATGAGTAGTTTTTTTTCCACAGGACTAACGAAGAACCAATGCATATACCAGTTTCCAAGTGTTTTTTTTT.GAAAAAATGGTTTAATATTTCTTAAAT   499
Consensus    aaatgagtagttttttttccacaggactaacgaagaaccaatgcatataccagtttccaagtgttttttttttgaaaaaatggtttaatatttcttaaat

BjuA032667   AGTTTACGAAAGTGTGTACTTGATTACCAGAAGACATATACTTTGGGATTATAACAATAAGGTCGGACAATTAACTATAGTGAATTAGTGATGATGATAT   600
BjABR1       AGTTTACGAAAGTGTGTACTTGATTACCAGAAGACATATACTTTGGGATTATAACAATAAGGTCGGACAATTAACTATAGTGAATTAGTGATGATGATAT   599
Consensus    agtttacgaaagtgtgtacttgattaccagaagacatatactttgggattataacaataaggtcggacaattaactatagtgaattagtgatgatgatat

BjuA032667   GCCAAAAGAATCTATCTCCCTCCATTTGCATAAAAGTCATTTTCCACTTGCATTTTGTTTACTACTTGTGCATTGCCCTTTGTCACCCATTTGGCCTAAT   700
BjABR1       GCCAAAAGAATCTATCTCCCTCCATTTGCATAAAAGTCATTTTCCACTTGCATTTTGTTTACTACTTGTGCATTGCCCTTTGTCACCCATTTGGCCTAAT   699
Consensus    gccaaaagaatctatctccctccatttgcataaaagtcattttccacttgcattttgtttactacttgtgcattgccctttgtcacccatttggcctaat

BjuA032667   CACTTTGTCGTAATCACTGAACACTCCTTTTGATTTTGTTTTGTTTTTAAGGACTTTCTCTTTATAATATCATTATTTGACTAGATTTAAAGAGAGTTAT   800
BjABR1       CACTTTGTCGTAATCACTGAACACTCCTTTTGATTTTGTTTTGTTTTTAAGGACTTTCTCTTTATAATATCATTATTTGACTAGATTTAAAGAGAGTTAT   799
Consensus    cactttgtcgtaatcactgaacactccttttgattttgttttgtttttaaggactttctctttataatatcattatttgactagatttaaagagagttat

BjuA032667   TAGTGAGCTAAAACAAAAGTTTGAATTTATATGTTTTATGTTTGTTTGTTTCCA                                                854
BjABR1       TAGTGAGCTAAAACAAAAGTTTGAATTTATATGTTTTATGTTTGTTTGTTTCCA                                                853
Consensus    tagtgagctaaaacaaaagtttgaatttatatgttttatgtttgtttgtttcca
```

B

```
BjuA032667   TCATCTTTTAAGTTTATTGAGGTTGGTAAACGTATAAAACATTTAGACCGGAGAAAGAAAAATCGAAAACGAAGAATATTTCCTGGGTCAAACGGTCGAA   100
BjABR1       .CATCTTTTAAGTTTATTGAGGTTGGCAAACGTATAAAACATTTAGACCGGAGAAAGAAAAATCGAAAACGAAGAATATTTCCTGGGTCAAACGGTCGAA    99
Consensus    tcatcttttaagtttattgaggttggcaaacgtataaaacatttagaccggagaaagaaaaatcgaaaacgaagaatatttcctgggtcaaacggtcgaa

BjuA032667   GACAAAATGAAAGACGAAGCTTGAAACAAGTGGGCTGTAGGTACGGCCCAGAAAAGGCATGTGCTTTGGCCTTTCACCACCACACCGTCCGAACGAAGCA   200
BjABR1       GACAAAATGAAAGACGAAGCTTGAAACAAGTGGGCTGTAGGTACGGCCCAGAAAAGGCATGTGCTTTGGCCTTTCACCACCACACCGTCCGAACGAAGCA   199
Consensus    gacaaaatgaaagacgaagcttgaaacaagtgggctgtaggtacggcccagaaaaggcatgtgctttggcctttcaccaccacaccgtccgaacgaagca

BjuA032667   TTTGACGTCGGCGTGAACCAACACCGGACTCGTGAGAGAACGCGCTTTTGTACAGCACGGCTTCTCTCTCAACTTGACCTTCAAGCTCACCGATTAGACA   300
BjABR1       TTTGACGTCGGCGTGAACCAACACCGGACTCGTGAGAGAACGCGCTTTTGTACAGCACGGCTTCTCTCTCAACTTGACCTTCAAGCTCACCGATTAGACA   299
Consensus    tttgacgtcggcgtgaaccaacaccggactcgtgagagaacgcgcttttgtacagcacggcttctctctcaacttgaccttcaagctcaccgattagaca

BjuA032667   AATAATAATATTTCATTTAATGGTTTTACCTATTTTTCAGCAGTTCATTCTTATA.TTAGTTATTAATCACCGAACCATAATATAATATTTAATATCAGT   399
BjABR1       AATAATAATATTTCATTTAATGGTTTTACCTATTTTTCAGCAGTTCATTCTTATAATTAGTTATTAATCACCGAACCATAATATAATATTTAATATCAGT   399
Consensus    aataataatatttcatttaatggttttacctatttttcagcagttcattcttataattagttattaatcaccgaaccataatataatatttaatatcagt

BjuA032667   GTCTTAATGTCTCCGTATTTTAGGAATGTTTGTATTCTTTTTGTTTTAAAAAGTAGTAGTATATGTTTTGGAATTTTCAAATCGATATAAAATCATTGTT   499
BjABR1       GTCTTAATGTCTCCGTATTTTAGGAATGTTTGTATTCTTTTTGTTTTAAAAAGTAGTAGTATATGTTTTGGAATTTTCAAATCGATATAAAATCATTGTT   499
Consensus    gtcttaatgtctccgtattttaggaatgtttgtattctttttgttttaaaaagtagtagtatatgttttggaattttcaaatcgatataaaatcattgtt

BjuA032667   TAAAGAAATTTTAGGAATTAGCAATTTAACTATCGTCTAATAAAAATACATAGAAAACGTAAAAATATAAGTTAATAACTTTTTTTATGCAACTTGTTTA   599
BjABR1       TAAAGAAATTTTAGGAATTAGCAATTTAACTATCGTCTAATAAAAATACATAGAAAACGTAAAAATATAAGTTAATAACTTTTTTTATGCAACTTGTTTA   599
Consensus    taaagaaattttaggaattagcaatttaactatcgtctaataaaaatacatagaaaacgtaaaaatataagttaataactttttttatgcaacttgttta

BjuA032667   ACTAAATAACACTATCATGCTATAGTGCTATAGAATCCCCCCCCCCCTCCCCGAGCTTAAACTTTACTTAATTAGATGTAGTCAATTAGGTTCGTTTAAG   699
BjABR1       ACTAAATAACACTATCATGCTATAGTGCTATAGAATCCCCCCCCCCCTCCCCGAGCTTAAACTTTACTTAATTAGATGTAGTCAATTAGGTTCGTTTAAG   699
Consensus    actaaataacactatcatgctatagtgctatagaatccccccccccctccccgagcttaaactttacttaattagatgtagtcaattaggttcgtttaag

BjuA032667   TTTTGATTAACAGTTAGCTGTTCACTTACATGTCGATGCAATTAAGTATCATGTCGATGCAATTAAGTATCACCCACAAGTTGCAAAAGTTTCAGATCCG   799
BjABR1       TTTTGATTAACAGTTAGCTGTTCACTTACATGTCGATGCAATTAAGTATCATGTCGATGCAATTAAGTATCACCCACAAGTTGCAAAAGTTTCAGATCCG   799
Consensus    ttttgattaacagttagctgttcacttacatgtcgatgcaattaagtatcatgtcgatgcaattaagtatcacccacaagttgcaaaagtttcagatccg

BjuA032667   TTTCATATAATCCAATTCATATTGACCAGAATCATCAAAACCAAGTTTCCTTTCGTTAGTATAGTTCAAATTTATAATATAAAGTTATTTACTTTATTTT   899
BjABR1       TTTCATATAATCCAATTCATATTGACCAGAATCATCAAAACCAAGTTTCCTTTCGTTAGTATAGTTCAAATTTATAATATAAAGTTATTTACTTTATTTT   899
Consensus    tttcatataatccaattcatattgaccagaatcatcaaaaccaagtttcctttcgttagtatagttcaaatttataatataaagttatttactttatttt

BjuA032667   ATAATATACTATATTTAAAACTTTAAAAGTGTATGTTTTACATTCACACGACCGAAACAGAGAGCAGCCGACTCTTTCATTTGGCGGCGCTGAGCTAAGA   999
BjABR1       ATAATATACTATATTTAAAACTTTAAAAGTGTATGTTTTACATTCACACGACCGAAACAGAGAGCAGCCGACTCTTTCATTTGGCGGCGCTGAGCTAAGA   999
Consensus    ataatatactatatttaaaactttaaaagtgtatgttttacattcacacgaccgaaacagagagcagccgactctttcatttggcggcgctgagctaaga

BjuA032667   CGAGGGTTTTACACAATAAAAATATTTCTATATCTATCAGCTTAAACCTTATTCTTTGTCTTAAGAAAAGAACATCTGATAAAACTCTCTCTCTCTCTCT  1099
BjABR1       CAAGGGTTTTACACAATAAAAATATTTCTATATCTATCAGCTTAAACCTTATTCTTTGTCTTAAGAAAAGAACATCTGATAAAACTCTCTCTCTCTCTCT  1099
Consensus    caagggttttacacaataaaaatatttctatatctatcagcttaaaccttattctttgtcttaagaaaagaacatctgataaaactctctctctctctct

BjuA032667   CTGCAGAGTTTTCTTTCCCTCTTAAGAAAAAAATAATATTTTTCTTTTTCTTTTTTTTATTTGTGGGTTTATAAGCTGCAAGTTTCAGACAAGGATTATA  1199
BjABR1       CTGCAGAGTTTTCTTTCCCTCTTAAGAAAAAAATAATATTTTTCCTTTTCTTTTTTTTATTTGTGGGTTTATAAGCTGCAAGTTTCAGACAAGGATTATA  1199
Consensus    ctgcagagttttctttccctcttaagaaaaaaataatatttttccttttcttttttttatttgtgggtttataagctgcaagtttcagacaaggattata

BjuA032667   AGGAGAGAATTG                                                                                          1211
BjABR1       AGGAGAGAATTG                                                                                          1211
Consensus    aggagagaattg
```
