# Supplementary material for: A tuber mustard AP2/ERF transcription factor gene, BjABR1, functioning in abscisic acid and abiotic stress responses, and evolutionary trajectory of the ABR1 homologous genes in Brassica species
Source: PeerJ. 2018 Dec 11;6:e6071. doi: 10.7717/peerj.6071 (PMC6294115; doi:10.7717/peerj.6071)
Supplement: Supplemental Information 7 — The partial sequence of the second exon of BniB045887-PA gene was signed by deep pink box and the rest of sequence was its downstream sequence of the coding region, while the third exon sequence of BjuB007684 was signed by red underline and the rest of sequence was its second intron sequence. Nucleotides conserved in two sequences are showed in dark gray. [file peerj-06-6071-s007.pdf]

```
BniB045887-PA  GTTGGTAATAATAATAACAACTATAAGAATAGTCCATCCTCATGATCGAACGGCTTTACTTCTTGTTTTTTTTTTCCTTTAGTATAGTGTTATGTTATAG   100
BjuB007684     GTTGGTAATAATAATAACAACTATAAGAATAGTCCATCCTCATGATCGAACGGCTTTACTTCTTGTTTTTTTTTTCCTTTAGTATAGTGTTATGTTATAG   100
Consensus      gttggtaataataataacaactataagaatagtccatcctcatgatcgaacggctttacttcttgttttttttttcctttagtatagtgttatgttatag

BniB045887-PA  AAAAGAGAAAAAAAAAAAAAGATTAGTTATAGCTTATTATAGTGTTTAGGTTTTTGTATTATTTTTTTTTCTTTTCAGTTTTATCATAATTTTGTATAAT   200
BjuB007684     ACAAGAGAAAAAA....AAAGATTAGTTATAGCTTATTATAGTGTTTAGGTTTTTGTATTATTTTTTTTTCTTTTCAGTTTTATCATAATTTTGTATAAT   196
Consensus      aaaagagaaaaaaaaaaaaagattagttatagcttattatagtgtttaggtttttgtattatttttttttcttttcagttttatcataattttgtataat

BniB045887-PA  AATTCGTTGTAAAAAA.TCATAAGGCAATAGTGGAAAAAATTAAGAGTTGTGTTTATGCTTTAAAAGGTTCCCTAATATCACACACAAAAGGGAAAGAAA   299
BjuB007684     AATTCGTTGTAAAAAAATCATAAGGAAATAGTGGAAAAAATTAAGAGTTGTGTTTATGCTTTAAAAGGTTCCCTAATATCACACACAAAAGGGAAAGAAA   296
Consensus      aattcgttgtaaaaaaatcataaggaaatagtggaaaaaattaagagttgtgtttatgctttaaaaggttccctaatatcacacacaaaagggaaagaaa

BniB045887-PA  AATTCGAAGGTATCATGTATGCGAACTGTGAAGTTGTGAACCAAACAAAGACAACAAAACTATACGAAAGAATTTTAGGACAGTGACAACAACTTGGCCC   399
BjuB007684     AATTCGAAGGTATCATGTATGCGAACTGTGAAGTTGTGAACCAAACAAAGACAACAAAACTATACGAAAGAATTTTAGGACAGTGACAACAACTTGGCCC   396
Consensus      aattcgaaggtatcatgtatgcgaactgtgaagttgtgaaccaaacaaagacaacaaaactatacgaaagaattttaggacagtgacaacaacttggccc

BniB045887-PA  ATTTAGGAAACACCTGGCCCATAAAGTTGATGTCTTTGGACTATATTGTTGCGGGAGATTTTGATTATGATTCTAAGAGCATCCACATTGGGGTTTATAG   499
BjuB007684     ATTTAGGAAACACCTGGCCCATAAAGTTGATGTCTTTGGACTATATTGTTGCGGGAGAATTTGATTATGATTGTAA.........................   472
Consensus      atttaggaaacacctggcccataaagttgatgtctttggactatattgttgcgggagaatttgattatgattctaagagcatccacattggggtttatag

BniB045887-PA  AGGAGGTTCACACAGAAATCAAGAAAAAAAAAAAAATCAATAAAATCATATGAACTTCTCTTTCCTAAAGTTCTTTCGGTTGAACCCCTCTCTCCTGAAGT   599
BjuB007684     ....................................................................................................   472
Consensus      aggaggttcacacagaaatcaagaaaaaaaaaaaaatcaataaaatcatatgaacttctctttcctaaagttctttcggttgaacccctctctcctgaagt

BniB045887-PA  TCATCACTGTAGCGTGGCCCCCACGTATCGTGGCGGCCCACGATTGGTCTAAGTTTTTTTTTTTTTAAAAAAACAAATTAAAAAGAAAAAGAAAAAAGTT   699
BjuB007684     ....................................................................................................   472
Consensus      tcatcactgtagcgtggcccccacgtatcgtggcggcccacgattggtctaagtttttttttttttaaaaaaacaaattaaaaagaaaaagaaaaaagtt

BniB045887-PA  TAATAATAAAAAATTAAAAAGATGAATCCCAAGAGGAAGTTCATTGACGTGGGTGCTCTAACTATCGTGGAAAAATTCAAGTAGGATTATGAATATTGAT   799
BjuB007684     ...............................................................CTATCGTGGAAGAATTCAAGTAGGATTATGAATATTGAT   511
Consensus      taataataaaaaattaaaaagatgaatcccaagaggaagttcattgacgtgggtgctctaactatcgtggaaaaattcaagtaggattatgaatattgat

BniB045887-PA  TGATAAAAGTTATTTTTAGTCAGGAAGGCCCTTTCTCAAAAAA.GTTATTTTTAGTCTAATCTGTGGATTATTCAAGAATAATTTCAGATCCCCCATCCC   898
BjuB007684     TGATAAAAGTTATTTTTAGTCAGGAAGGCCCTTTCTCAAAAAAAGTTATTTTTAGTCTAATCTGTGGATTATTCAAGAATAATTTCAGATCCCCCATCCC   611
Consensus      tgataaaagttatttttagtcaggaaggccctttctcaaaaaaagttatttttagtctaatctgtggattattcaagaataatttcagatcccccatccc

BniB045887-PA  AAATATTTTTCTGGTTATCGTCCAATTATTTTTTAGAGAATTTGCTAACTTGTCTTATATGTACCGTAATATTATGACATGCAATATTCATATATATACA   998
BjuB007684     AAATATTTTTCTGGTTATCGTCCAATTATTTTTTAGAGAATTTGCTAACTTGTCTTATATGTACCGTAATATTATGACATGCAATATTCATATATATACA   711
Consensus      aaatatttttctggttatcgtccaattattttttagagaatttgctaacttgtcttatatgtaccgtaatattatgacatgcaatattcatatatataca

BniB045887-PA  CTTCATTTTTTGTCTTGCGAAAATACTATGAAAGATA.GATTTTCTTTTGGCATCATGAAAGATAAGATTACTGTACTGTTAAATTCCGTATATAAAATA  1097
BjuB007684     CTTCATTTTTTGTCTTGCGAAAATACTATGAAAGATAAGATTTTCTTTTGGCATCATGAAAGATAAGATTACTGTACTGTTAAATTCCGTATATAAAATA   811
Consensus      cttcattttttgtcttgcgaaaatactatgaaagataagattttcttttggcatcatgaaagataagattactgtactgttaaattccgtatataaaata

BniB045887-PA  AATTTTGTGATTTTTAAAACATTTACATGAACGGATGAGTATTCTCTAAATCTCTAATATTATTTTCTTACACTAATTCTCAGGAGAATGCGTAATATAA  1197
BjuB007684     AATTTTGTGATTTTTAAAACATTTACATGAACGGATGAGTATTCTCTAAATCTCTAATATTATTTTCTTACGCTAATTCTCAGGAGAATGCGTAATATAA   911
Consensus      aattttgtgatttttaaaacatttacatgaacggatgagtattctctaaatctctaatattattttcttacactaattctcaggagaatgcgtaatataa

BniB045887-PA  TGACAACAAAAGAAAACATAGAAAGAGATGAGAGAATCTCTTTTCGTCTCTTTTCTCTCTTGTGACTTCTCATTTCTATGTATCAACATTTTGTTTTCCG  1297
BjuB007684     TGACAACAAAAGAAAACATAGAAAGAGATGAGAGAATCTCTTTCCGTCTCTTTTCTCTCTTGTGACTTCTCATTTCTATGTATCAACATTTTGTTTTCCG  1011
Consensus      tgacaacaaaagaaaacatagaaagagatgagagaatctctttccgtctcttttctctcttgtgacttctcatttctatgtatcaacattttgttttccg

BniB045887-PA  TGAGTGAATTAGTTAGATAAGCTTTAATTACACTTTTCGGTGATGTATAAACCTTATTTATCATCAAAAGTTTTTTAATTTTTTTTT.CCTTGACCCATGT  1396
BjuB007684     TGAGTGAATTAGTTAGATAAGCTTTAATTACACTTTTCGGTGATGTATAAACCTTATTTATCATCAAAAGTTTTTTATTTTTTTTTTTCCTTGACCCATGT  1111
Consensus      tgagtgaattagttagataagctttaattacacttttcggtgatgtataaaccttatttatcatcaaaagttttttaattttttttttccttgacccatgt

BniB045887-PA  AGATGCAATAGGTTTTGTTTGGTTCTAATTAGGAATCAAGCATAGATTTGTGACTTTTCAAATCTTGGTTTTTTGATGAAATGTTTTAATGCAAGTATTA  1496
BjuB007684     AGATGCAATAGGTTTTGTTTGGTTCTAATTAGGAATCAAGCATAGATTTGTGACTTTTCAAATCTTGGTTTTTTAATGAAATGTTTTAATGCAAGTATTA  1211
Consensus      agatgcaataggttttgtttggttctaattaggaatcaagcatagatttgtgacttttcaaatcttggttttttaatgaaatgttttaatgcaagtatta

BniB045887-PA  TAACTGACAATATATGAAGTATTTTTACAAGAAATGAAGTTTCATGCTACAAAAGAAAGACTAATTCTATTCATCGTTTGATTGTCATAAACAATATTTG  1596
BjuB007684     TAACTGACAATATATGAAGTATTTTTACAAGAAATGAAGTTTCATGCTACAAAAGAAAGACTAATTCTATTCATCGTTTGATTGTCATAAACAATATTTG  1311
Consensus      taactgacaatatatgaagtatttttacaagaaatgaagtttcatgctacaaaagaaagactaattctattcatcgtttgattgtcataaacaatatttg

BniB045887-PA  TGCACAAAAATAGATATATACAAGGCGTCATCGGTCTCCATTTCTTATATGTGGAGTTCTTCCGCATTTCTTATATGTAAATTTTAAGGCACGATTAGCA  1696
BjuB007684     TGCACAAAAATAGATATATACAAGGCGTCATCGGTCTCCATTTCTTATATGTGGAGTTCTTCCGCATTTCTTATATGTAAATTTTAAGGCACGATTAGCA  1411
Consensus      tgcacaaaaatagatatatacaaggcgtcatcggtctccatttcttatatgtggagttcttccgcatttcttatatgtaaattttaaggcacgattagca

BniB045887-PA  GCAGTTGATTTCCATCTGGTTCTAACTACAGCCATAGCGCTGTTGGCGTTGGTGAAACTCTGTCTGAGCTGCTCTCTGCAAGCG.TCGCCGTCCTCAACC  1795
BjuB007684     GCAGTTGATTTCCATCTGGTTCTAACTACAGCCATAGCGCTGTTGGCGTTGGTGAAACTCTGTCTGAGCTGCTCTCTGCAAGCGGTCGCCGTCCTCAACC  1511
Consensus      gcagttgatttccatctggttctaactacagccatagcgctgttggcgttggtgaaactctgtctgagctgctctctgcaagcggtcgccgtcctcaacc

BniB045887-PA  GGGACCGATCTCTCAAAGCAGTCGCCGTCCTGAGCCGGGACCGATCCCGCAATATCTTGGTACTCCAAGCGGTCCAACACCACTA  1880
BjuB007684     GGGACCGATCTCTTAAAGCAGTCGCCGTCCTGAGCCGGGACCGATCCCGCAATATCTTGGTACTCCAAGCGGTCCAACACCACTA  1596
Consensus      gggaccgatctctcaaagcagtcgccgtcctgagccgggaccgatcccgcaatatcttggtactccaagcggtccaacaccacta
```
