# Supplementary material for: A tuber mustard AP2/ERF transcription factor gene, BjABR1, functioning in abscisic acid and abiotic stress responses, and evolutionary trajectory of the ABR1 homologous genes in Brassica species
Source: PeerJ. 2018 Dec 11;6:e6071. doi: 10.7717/peerj.6071 (PMC6294115; doi:10.7717/peerj.6071)
Supplement: Supplemental Information 9 — (A) Sequence alignment between the intron of Bo9g018330.1 and the second intron of GSBRNA2T00134741001. (B) Sequence alignment between the first exon of Bo9g018330.1 and the second exon of GSBRNA2T00134741001. (C) the second exon of Bo9g018330.1 and the third exon of GSBRNA2T00134741001. Nucleotides conserved in two sequences are showed in dark gray. [file peerj-06-6071-s009.pdf]

A

```
Bo9g018330.1          GTAAATTTTTTTCATTTTTATATAATAACACGCGTGCACCATCAGATTAAGTTTGAATTATCTCACGTGTATGTGTATATAAAAGAATGGTGGATTCTCA    100
GSBRNA2T00134741001   GTAATTTTTTTTCATTTTTATATAATAACACGCGTGCACCATCAGATTAAGTTTGAATTATCTCACGTGTATGTGTATATAAAAGAATGGTGGATTCTCA    100
Consensus             gtaaatttttttcatttttatataataacacgcgtgcaccatcagattaagtttgaattatctcacgtgtatgtgtatataaaagaatggtggattctca

Bo9g018330.1          AAACACAAAAACTATTTTTCCTTCAATTTTTTCAAAACTTCAAATGAATCGTGAGATTTTGAAACCATTTATGATATAATGTTTTAATAAATTTGTGATG    200
GSBRNA2T00134741001   AAACACAAAAACTATTTTTCCTTCAATTTTTTCAAAACTTCAAATGAATCGTGAGATTTTGAAACCATTTATGATAAAATGTTTTAATAAATTTGTGATG    200
Consensus             aaacacaaaaactatttttccttcaattttttcaaaacttcaaatgaatcgtgagattttgaaaccatttatgataaaatgttttaataaatttgtgatg

Bo9g018330.1          TGGTAAACTATATACTCCCTCAGTTCTTTAATGATAGATTTTTAGAAAAAAAAATTTGTTTCACAAAAATGTATTTTTTTTGTGTTTTCTATAAAAAAAT    300
GSBRNA2T00134741001   TGGTAAACTATATACTCCCTCAGTTCTTTAATGATAGTCTTTTTAGAAAAAAAAATTTGTTTCACAAAA...TGTATTTTTTTGTATTTTCTATAAAAAAAT    297
Consensus             tggtaaactatatactccctcagttctttaatgatagactttttagaaaaaaaaatttgtttcacaaaaatgtatatttttttgtattttctataaaaaaat

Bo9g018330.1          TGTAAACTTCAAGAAAATCAATTAACTTTATTGAATTACTATTGGTTAAAAGTTATTGAAAATTGAAAATTACAGAAAACGATACATTTATTATGGTAGT    400
GSBRNA2T00134741001   TGTAAACTTCAAGAAAATTAATTGACTTTCTTGAATTACTATTGGTTAAAAGTTATTGAAAATTGAAAATTACAGAAAACGATACATTTATTATGGTAGT    397
Consensus             tgtaaacttcaagaaaatcaattaactttattgaattactattggttaaaagttattgaaaattgaaaattacagaaaacgatacatttattatggtagt

Bo9g018330.1          TTAATGTGTTTTCTTAATATGTGTGAAAATAGTAAAAAGTCTATTTTTGTGAAACAGAGGGAGGTAGTATATACCAAGTGTATGAGTCACATCATATAAA    500
GSBRNA2T00134741001   TTAATGTGTTTTTTTAATACGTGTGAAAATAGTAAAAAATCTATTTTTGTGAAACAGAGGGAGAGAGTATATACCTAGTGTATGAGTCACATCATATCAA    497
Consensus             ttaatgtgttttcttaatacgtgtgaaaatagtaaaaaatctatttttgtgaaacagagggagagagtatataccaagtgtatgagtcacatcatataaa

Bo9g018330.1          ACACAACATCTAAATATTTGTTCTTTTGTTGTGTCTTTATTTGTTTAATTTTAATGAAAAGTTGCTCTTTTGTTGCACTCATGCAATATACAAAACAACA    600
GSBRNA2T00134741001   ACACAACATCTAAATATTTGCTCTTTTGTTGTGTTTTTATTTGTTTAATTTTAATGAAAAGTTGCTCTTTTGTTGCACTCATGCAATATAAAAAACAACA    597
Consensus             acacaacatctaaatatttgctcttttgttgtgtctttatttgtttaattttaatgaaaagttgctcttttgttgcactcatgcaatataaaaaacaaca

Bo9g018330.1          TAAAGAATAATCACAATGTTTTTTTTT.GGTTTCCTCTAGAAATTCCATGAGTTCTTTCATAAATGAGTTTTTTTTTCACAAGACTATCGAAGAACCAA    699
GSBRNA2T00134741001   TAAAGAATAATCACAATGTTTTTTTTTTGGTTTCCTCTAGAAATTCCATGAGTTCTTTCATAAATGAGTTTTTTTTTCACAAGACTATCGAAGAACCAA    697
Consensus             taaagaataatcacaatgttttttttttggtttcctctagaaattccatgagttctttcataaatgagtttttttttcacaagactatcgaagaaccaa

Bo9g018330.1          TGCATATAACCAGTTTCCAAGTGATTTTTTTTTGTTTTGAAAAAA.TGGTTTAATATTTCATAAATAGTTTACGAAAGTGTACTTGATTACCAGAAGACA    798
GSBRNA2T00134741001   TGCATATAACCAGTTTCCAAGTGATTTTTTTTTGTTTTGAAAAAAATGGTTTAATATTTCTTAAATAGTTTACGAAAGTGTACTTGATTACCAGAAGACA    797
Consensus             tgcatataaccagtttccaagtgatttttttttgttttgaaaaaaatggtttaatatttcataaatagtttacgaaagtgtacttgattaccagaagaca

Bo9g018330.1          TATACTTTGGGATTACAATAAGGTCGGACAATTAACTATAGTGATGATGATATGCCAAAAGAATCTATCTCCCTCCATTTGCATAAAGAGTCCTTTTCCA    898
GSBRNA2T00134741001   TATACTTTGGGATTACAATAAGGTCGGACAATTAACTATAGTGATGATGATATGCCAAAAGAATCTATCTCCCTCCATTTGCATAAAGAGTCCTTTTCCA    897
Consensus             tatactttgggattacaataaggtcggacaattaactatagtgatgatgatatgccaaaagaatctatctccctccatttgcataaagagtccttttcca

Bo9g018330.1          CTTGCCTTTTGTTTACTACTTGTTCATTGCCCTTTGTCACCCATTTGGCCTAATCACTTTGTCGTAATCACTGAACACTCTTTTTGATTTTGTTTTGTTT    998
GSBRNA2T00134741001   CTTGCCTTTTGTTTACTACTTGTTCATTGCCCTTTGTCACCCATTTGGCCTAATCACTTTGTCGTAATCACTGAACACTCTTTTTGATTTTGTTTTGTTT    997
Consensus             cttgccttttgtttactacttgttcattgccctttgtcacccatttggcctaatcactttgtcgtaatcactgaacactctttttgattttgttttgttt

Bo9g018330.1          TTAAGGATTTTCTCTTTTTTTTTTTTTGTCAAACAAGGATTTTCTCTTTATAATATCATTATTTGAGTAGATTTAAAGAGAGTTATTAGTGAGCCAAAAC    1098
GSBRNA2T00134741001   TTAAGGATTTTCTCTTTTTTTTTTT..TGTCAAACAAGGATTTTCTCTTTATAATATCATTATTTGAGTAGATTTAAAGAAAGTTATTAGTGAGCCAAAAC    1095
Consensus             ttaaggattttctctttttttttttttgtcaaacaaggattttctctttataatatcattatttgagtagatttaaagaaagttattagtgagccaaaac

Bo9g018330.1          AAAAGTTTGAATTTATATGTTTTGTGTTTGTTTGTTTCCA                                                                1138
GSBRNA2T00134741001   AAAAGTTTGAATTTATATGTTTTGTGTTTGTTTGTTTCAA                                                                1135
Consensus             aaaagtttgaatttatatgttttgtgtttgtttgtttcaa
```

B

```
Bol015899             ............ATGTGTGCCTTAAAAGTGGCAAATCAGGAAGATAACGTTGGTAAAAAAGCCGAGCCTACCACTCGTGAAGATGATGATCACCGGACGT    88
GSBRNA2T00134741001   GGAGAGAATTGTGTGTGTGCCTTAAAAGTGGCAAATCAGGAAGATAACGTTGGTAAAAAAGCCGAGCCTACCACTCGTGAAGATGATGATCACCGGACGT    100
Consensus             ggagagaattgtatgtgtgccttaaaagtggcaaatcaggaagataacgttggtaaaaaagccgagcctaccactcgtgaagatgatgatcaccggacgt

Bol015899             TATCTGACATCGATCAATGGCTTTACTCATTTTCGGCGGAAGATGACCACCTCCACCGTCATAACTCCCTCACGCCTCCTTCCTCGTCTCTTAGTATTAG    188
GSBRNA2T00134741001   TATCTGACATCGATCAATGGCTTTACTCATTTTCGGCGGAAGATGACCACCTCCACCGTCATAACTCCCTCACGCCTCCTTCCTCGTCTCTTAGTATTAG    200
Consensus             tatctgacatcgatcaatggctttactcattttcggcggaagatgaccacctccaccgtcataactccctcacgcctccttcctcgtctcttagtattag

Bol015899             CAGAGAGAAAGAGATGTCTGCAATCGTCTCTGCTTTGACACATGTCGTCGCTGGAAATGTTCCTACTCGTCAATACGGTGGAGAAGGGACGTCGAATTCT    288
GSBRNA2T00134741001   CAGAGAGAAAGAGATGTCTGCAATCGTCTCTGCTTTGACACATGTCGTCGCTGGAAATGTTCCTACTCGTCAATACGGTGGAGAAGGGACGTCGAATTCT    300
Consensus             cagagagaaagagatgtctgcaatcgtctctgctttgacacatgtcgtcgctggaaatgttcctactcgtcaatacggtggagaagggacgtcgaattct

Bol015899             TCTTCTTCCCCCGGCCAGAAAAGGAGGAGAGAGGTGGAGGAAGGTGGCGGCGGTGGCAAAGACGTTAAGGCAGCTAATACTTTGACCGTTGATCAATATT    388
GSBRNA2T00134741001   TCTTCTTCCCCCGGCCAGAAAAGGAGGAGAGAGGTGGAGGAAGGTGGCGGTGGTGGCAAAGACGTTAAGGCAGCTAATACTTTGACCGTTGATCAATATT    400
Consensus             tcttcttcccccggccagaaaaggaggagagaggtggaggaaggtggcggcggtggcaaagacgttaaggcagctaatactttgaccgttgatcaatatt

Bol015899             TCTCAGGCGGTAGTTCTAGTTTCAAA                                                                              414
GSBRNA2T00134741001   TCTCAGGCGGTAGTTCTAGTTCCAAA                                                                              426
Consensus             tctcaggcggtagttctagttccaaa
```

C

```
GSBRNA2T00134741001   TGGGAGAAGCTTCGAGCAACATGTCAGGTTCAGTCCCAACATATGAATACACAACAACGCCAAATGTCAATACAGAAACGTTGTTGTTAAGTGGGGACGG    100
Bol015899             TGGGAGAAGCTTCGAGCCACATGTCAGGTTTAGTCCCAACATATGAATACACAACAACGCCAAATGTCAATACAGAAACGTTGTTGTTAAGTGGGGACGG    100
Consensus             tgggagaagcttcgagcaacatgtcaggttcagtcccaacatatgaatacacaacaacgccaaatgtcaatacagaaacgttgttgttaagtggggacgg

GSBRNA2T00134741001   ACCTCAAAGAAGATACAGAGGAGTGAGACAAAGACCGTGGGGAAAATGGGCGGCTGAGATTCGAGATCCTTTCAAAGCGGCTAGAGTTTGGCTCGGTACA    200
Bol015899             ACCTCAAAGAAGATACAGAGGAGTGAGACAAAGACCGTGGGGAAAATGGACGGCTGAGATTCGAGATCCTTTCAAAGCGGCTAGAGTTTGGCTCGGTACA    200
Consensus             acctcaaagaagatacagaggagtgagacaaagaccgtggggaaaatggacggctgagattcgagatcctttcaaagcggctagagtttggctcggtaca

GSBRNA2T00134741001   TTTGACAATGCTGAATCAGCTGCAAGAGCTTATGACGAAGCTGCTCTTCGGTTTAGAGGCAACAAAGCCAAACTAAACTTCCCTGAAAACGTTAAACTAG    300
Bol015899             TTTGACAATGCTGAATCAGCTGCAAGAGCTTATGACGAAGCTGCTCTTCGGTTTAGAGGCAACAAAGCCAAACTAAACTTCCCTGAAAACGTTAAACTAG    300
Consensus             tttgacaatgctgaatcagctgcaagagcttatgacgaagctgctcttcggtttagaggcaacaaagccaaactaaacttccctgaaaacgttaaactag

GSBRNA2T00134741001   TTCGACCAGCTTCAATCGCCCCAACACTATCTGTACATCAAACAACTGTTCAGAGACCAACGCAGTTAAGAAACTCGGGTTCTACAAGTACCCTTTTGCC    400
Bol015899             TTCGACCAGCTTCAACCGCCCCAACACTATCTGTACAGCAAACAACTGTTCAGAGACCAACACAGTTAAGAAACTCGGGTTCTACAAGTACCCTTTTGCC    400
Consensus             ttcgaccagcttcaaccgccccaacactatctgtacagcaaacaactgttcagagaccaacacagttaagaaactcgggttctacaagtacccttttgcc

GSBRNA2T00134741001   CGTAAGACATGCTTCGGATCAAAACGTTCATTCTCAGCTGTTGATGCAATCTTACAACTTCAGTCACTTGGAGTTGGCTCATCACCAACAACAATTTCAG    500
Bol015899             CGTAAGACATGCTTCGGATCAAAACGTTCATTCTCAGCTGTTGATGCAATCTTACAACTTCAGTCACTTGGAGTTGGCTCATCACCAACAACAATTTCAG    500
Consensus             cgtaagacatgcttcggatcaaaacgttcattctcagctgttgatgcaatcttacaacttcagtcacttggagttggctcatcaccaacaacaatttcag

GSBRNA2T00134741001   CATCAACAACAATCTTTATATGATCAAGTGTCATTTCCATTGCGTTTTGGTCACACCGGAGGCTCAACGATGCAACCTACATCAACTTCTTCGTCTCGTT    600
Bol015899             CATCAACAGCAATCTTTATATGATCAAGTGTCATTTCCATTGCGTTTTGGTCACACCGGAGGCTCAACGATGCAACCTACATCAACTTCTTCGTCTCGTT    600
Consensus             catcaacaacaatctttatatgatcaagtgtcatttccattgcgttttggtcacaccggaggctcaacgatgcaacctacatcaacttcttcgtctcgtt

GSBRNA2T00134741001   CTACTTCTATGTTTTCTCCGGCAGAAGTTCAACCAAAGCTAGAATCAGCTAGCGAAACCGATCGTCTACAGGATCTACAGTCATTAGGCAAGGCTAGTAA    700
Bol015899             CTACTTCTATGTTTTCTCCGGCAGCAGTTCAACCAAAGCTAGAATCAGCTAGCGAAACCGATCGTCTACAGGATCTACAATCATTAGGCAAGGCTAGTAA    700
Consensus             ctacttctatgttttctccggcagaagttcaaccaaagctagaatcagctagcgaaaccgatcgtctacaggatctacaatcattaggcaaggctagtaa

GSBRNA2T00134741001   TAATAATAACAACTATAATAATAGCCCATCCTCTTA                                                                    736
Bol015899             TAATAATAACAACTATAATAATAGCCCATCCTCTTA                                                                    736
Consensus             taataataacaactataataatagcccatcctctta
```
