# Supplementary material for: A tuber mustard AP2/ERF transcription factor gene, BjABR1, functioning in abscisic acid and abiotic stress responses, and evolutionary trajectory of the ABR1 homologous genes in Brassica species
Source: PeerJ. 2018 Dec 11;6:e6071. doi: 10.7717/peerj.6071 (PMC6294115; doi:10.7717/peerj.6071)
Supplement: Supplemental Information 10 — The first exon sequence of GSBRNA2T00134741001 gene was signed by red box. Nucleotides conserved in two sequences are showed in dark gray. [file peerj-06-6071-s010.pdf]

```
GSBRNA2T00134741001   ATGATCAAACGAAACCGGGAGAACCCTG..GTGATGGCCGGGAAGACGATGTGACGGAATCATCCCTTCTCAGAAACAAAAGCTGGCGAAGAAGTGTTGT    98
Bol015899             ATGATCAAACGAAACCGGGAGAACCCCGCGGTGATGGCCGGGAAGACGATGTGACGGAATCATCCCTTCTCGGAGACAAAAGCCGGCGAAGAAGTGTTGT   100
Consensus             atgatcaaacgaaaccgggagaaccccgcggtgatggccgggaagacgatgtgacggaatcatcccttctcagaaacaaaagccggcgaagaagtgttgt

GSBRNA2T00134741001   GAGAACCACCACTTCTTGGAGTCAAAAGCCCAAACTTTCGGAAGACTTAACCGGAATAATCCTGTGATGGGAACAAGTCGGCGAAAACTCCCCCGAAAGA   198
Bol015899             GAGAACCACCACTTCCTGGAGTCAAAAGCCCGAACCTTCGAAAGACTTAACCGGAATAATCCGGTGATGGGAACAAGTCGGCAAAAACTCCTCTGAAAGA   200
Consensus             gagaaccaccacttcctggagtcaaaagcccaaaccttcgaaagacttaaccggaataatccggtgatgggaacaagtcggcaaaaactcccccgaaaga

GSBRNA2T00134741001   TTGAATTTTGATAAGAAGAAGAGAAATTGAGAAAGAGAGAAGCCGACACAATCATCAAAACCAAGTTTCCTTTCGTTATGATAAAAAGTTATTTACTTTA   298
Bol015899             TTGAATTTTGATAAGAAGAAGAGAAATTGAGAAAGAGAGAAGCCGACACAATCATCAAAACCAAGTTTCCTTTCGTTATGATATAAAGTTATTTACTTTA   300
Consensus             ttgaattttgataagaagaagagaaattgagaaagagagaagccgacacaatcatcaaaaccaagtttcctttcgttatgataaaaagttatttacttta

GSBRNA2T00134741001   TTTATAATTTACTATATTTAAAACTTTAAAAGTGTATGTTTTACATTCACACGACCGAAACAGAGAGCAGCCGACTCTTTCATTTGGCGGCGCTGAGCTA   398
Bol015899             TTTATAATTTACTATATTTAAAACTTTAAAAGTGTATGTTTTACATTCACACGACCGAAACAGAGAGCAGCCGACTCTTTCATTTGGCGGCGCTGAGCTA   400
Consensus             tttataatttactatatttaaaactttaaaagtgtatgttttacattcacacgaccgaaacagagagcagccgactctttcatttggcggcgctgagcta

GSBRNA2T00134741001   AGAAAAGGGTTTTACACGATAAAAATATTTCTATATCTATCAGCTTAAACCTTATTCTTTGTCTTAAGAACATCTGATAAAACTCTGTCTCTCTCAACAG   498
Bol015899             AGAAAAGGGTTTTACACGATAAAAATATTTCTATATCTATCAGCTTAAACCTTATTCTTTGTCTTAAGAACATCTGATAAAACTCTGTCTCTCTCAACAG   500
Consensus             agaaaagggttttacacgataaaaatatttctatatctatcagcttaaaccttattctttgtcttaagaacatctgataaaactctgtctctctcaacag

GSBRNA2T00134741001   AGATTTCTTTCCCTCTAAGAAAAAAATAATAATTTCTTTTTCTTTTTTTATTTGTGGGTTTATAAGCTTCAAGTTTCAGACAAGGATTATAG.........   589
Bol015899             AGATTTCTTTCCCTCTAAGAAAAAAATAATAATTTCTTTTTCTTTTTTTATTTGTGGGTTTATAAGCTTCAAGTTTCAGACAAGGATTATAGGGAGAGAAT   600
Consensus             agatttctttccctctaagaaaaaaataataatttctttttctttttttatttgtgggtttataagcttcaagtttcagacaaggattatagggagagaat

GSBRNA2T00134741001   ..                                                                                                      589
Bol015899             TG                                                                                                      602
Consensus             tg
```
